# Protection from environmental enteric dysfunction and growth improvement in malnourished newborns by amplification of secretory IgA

**DOI:** 10.1016/j.xcrm.2024.101639

**Published:** 2024-07-02

**Authors:** Lisa Perruzza, Tanja Rezzonico Jost, Matteo Raneri, Giorgio Gargari, Martina Palatella, Benedetta De Ponte Conti, Frauke Seehusen, Julia Heckmann, Dorothee Viemann, Simone Guglielmetti, Fabio Grassi

**Affiliations:** 1 Institute for Research in Biomedicine, Faculty of Biomedical Sciences, Università della Svizzera Italiana, 6500 Bellinzona, Switzerland; 2 Division of Food Microbiology and Bioprocesses, Department of Food, Environmental and Nutritional Sciences (DeFENS), University of Milan, 20133 Milan, Italy; 3 Graduate School of Cellular and Molecular Sciences, University of Bern, 3012 Bern, Switzerland; 4 Institute of Veterinary Pathology, Vetsuisse Faculty, University of Zurich, 8057 Zurich, Switzerland; 5 Department of Pediatrics, University Hospital Würzburg, 97080 Würzburg, Germany; 6 Cluster of Excellence RESIST (EXC 2355), Hannover Medical School, 30625 Hannover, Germany; 7 Center for Infection Research, University Würzburg, 97080 Würzburg, Germany; 8 Department of Biotechnology and Biosciences (BtBs), University of Milano-Bicocca, 20126 Milan, Italy

**Keywords:** malnutrition, environmental enteric dysfunction, microbiota, breast milk, secretory immunoglobulin, live biotherapeutic, purinergic signaling, mucosal immunity, T follicular helper cell

## Abstract

Environmental enteric dysfunction (EED) is a condition associated with malnutrition that can progress to malabsorption and villous atrophy. Severe EED results in linear growth stunting, slowed neurocognitive development, and unresponsiveness to oral vaccines. Prenatal exposure to malnutrition and breast feeding by malnourished mothers replicates EED. Pups are characterized by deprivation of secretory IgA (SIgA) and altered development of the gut immune system and microbiota. Extracellular ATP (eATP) released by microbiota limits T follicular helper (Tfh) cell activity and SIgA generation in Peyer’s patches (PPs). Administration of a live biotherapeutic releasing the ATP-degrading enzyme apyrase to malnourished pups restores SIgA levels and ameliorates stunted growth. SIgA is instrumental in improving the growth and intestinal immune competence of mice while they are continuously fed a malnourished diet. The analysis of microbiota composition suggests that amplification of endogenous SIgA may exert a dominant function in correcting malnourishment dysbiosis and its consequences on host organisms, irrespective of the actual microbial ecology.

## Highlights

• Malnourishment of prospective dams replicates EED in pups
• Malnourished mothers and offspring are characterized by SIgA impoverishment
• An apyrase-releasing live biotherapeutic induces SIgA amplification in pups
• SIgA amplification improves pups’ growth and intestinal immune competence

## Graphical abstract

**Figure:**
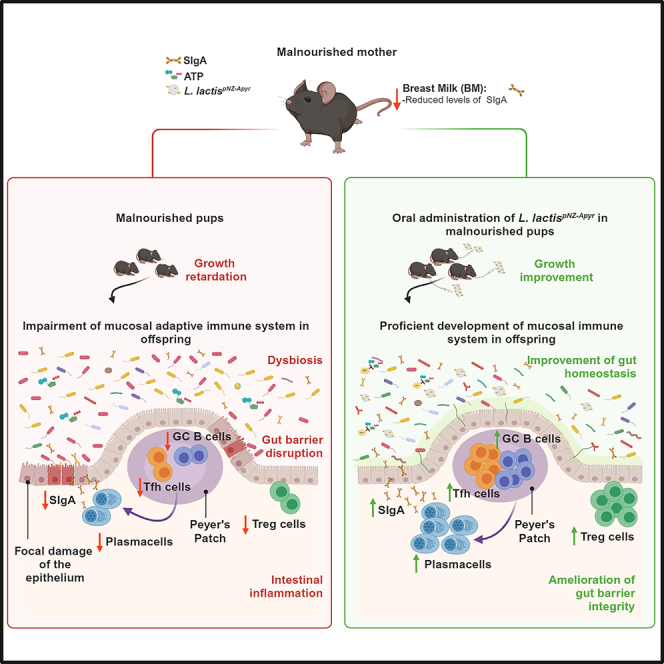

## Teaser

Environmental enteric dysfunction (EED) is a causal factor of stunting and neurocognitive defects in malnourished infants. Perruzza et al. develop a bioengineered live biotherapeutic able to restore intestinal immune functions in offspring from malnourished dams. The resulting amplification of mucosal secretory IgA ameliorates EED and partially restores pups’ growth.

## Introduction

Poor sanitary conditions and malnutrition profoundly affect human organism’s physiology and compromise development in early childhood. In 2022, almost 150 million children under 5 years of age were affected by stunting due to living in impoverished environments, Sub-Saharan Africa and Southern Asia comprising more than two-third of affected children.^1^ Restoration of nutrition and amelioration of sanitary settings are usually not sufficient to correct the pathological traits resulting from these situations, particularly stunting.^2^ Environmental enteric dysfunction (EED) is a disease of the small intestine characterized by diarrhea and malabsorption originally described in individuals exposed to high levels of fecal-oral contamination.^3^ It has been associated to malnourished (MAL) children and is characterized by severe intestinal malabsorption and villous atrophy in the small bowel^4^^,^^5^^,^^6^; these features have been related to mucosal inflammation and compromised intestinal barrier integrity.^7^^,^^8^ In children in low- and middle-income countries (LMICs), EED has been associated to linear growth stunting,^9^^,^^10^ slowed neurocognitive development,^11^ and reduced responsiveness to oral vaccines.^12^ Understanding the pathogenetic mechanisms underlying EED will allow implementing appropriate therapeutic approaches for these children. Recently, a causal relationship has been established in stunted children between microbiota structure in the duodenum, EED pathogenesis and growth stunting,^13^ thereby suggesting a rationale for targeting the small intestinal microbiota to ameliorate EED. Accordingly, promoting the restoration of gut bacterial composition in MAL children by specific complementary food beneficially influenced growth.^14^

At the time of delivery, the newborn enters in contact with vaginal and fecal microbes of maternal origin.^15^ Thereafter, the rapid rise in bacterial colonization of the intestine is characterized by the presence of age-specific bacterial species. Initially, mucosal colonization is characterized by fluctuating changes in microbial diversity until a relatively stable homeostatic condition is reached throughout adulthood.^16^ Breastfeeding is important in shaping the development of a narrow microbiota dominated by species metabolizing milk oligosaccharides. In fact, the adult-like bacterial density in the human intestinal tract is reached few days after birth^17^; however, it takes 2–3 years in humans^18^ and 3–4 weeks in mice^19^ to reach the adult-like bacterial diversity/richness. The relative variability and instability of the neonatal microbial community structure render the intestine more sensitive to colonization by environmental pathobionts.^20^ In this respect, maternal milk plays a fundamental role in feeding a physiological microbial community and conditioning the intestinal immune system to adapt commensal bacteria to the development of the whole organism. Early exposure to maternal secretory IgA (SIgA) in breast milk ensures the long-term integrity of the intestinal barrier and shapes the physiological microbiota composition, thereby promoting intestinal homeostasis. Both passive SIgA in breast milk and endogenous SIgA production are required to limit the expression of inflammatory genes in epithelial cells that are associated to an increased risk of developing intestinal inflammatory diseases.^21^

To comprehensively address the consequences of malnutrition on the developmental programming of the intestinal ecosystem starting from intrauterine life, we established an intergenerational model of malnutrition. Female mice were exposed to impoverished diet before breeding, during pregnancy and breast feeding, and applied to offspring toward adulthood. The impoverishment of SIgA in the breast milk of MAL dams might contribute to defective development of the endogenous mucosal adaptive immune system in offspring. We show that amplification of endogenous SIgA in newborns from MAL mothers can be exploited to correct growth stunting and immune dysfunction resulting from an MAL diet.

## Results

### Prenatal exposure to malnutrition results in EED features in the offspring

**Figure 1:**
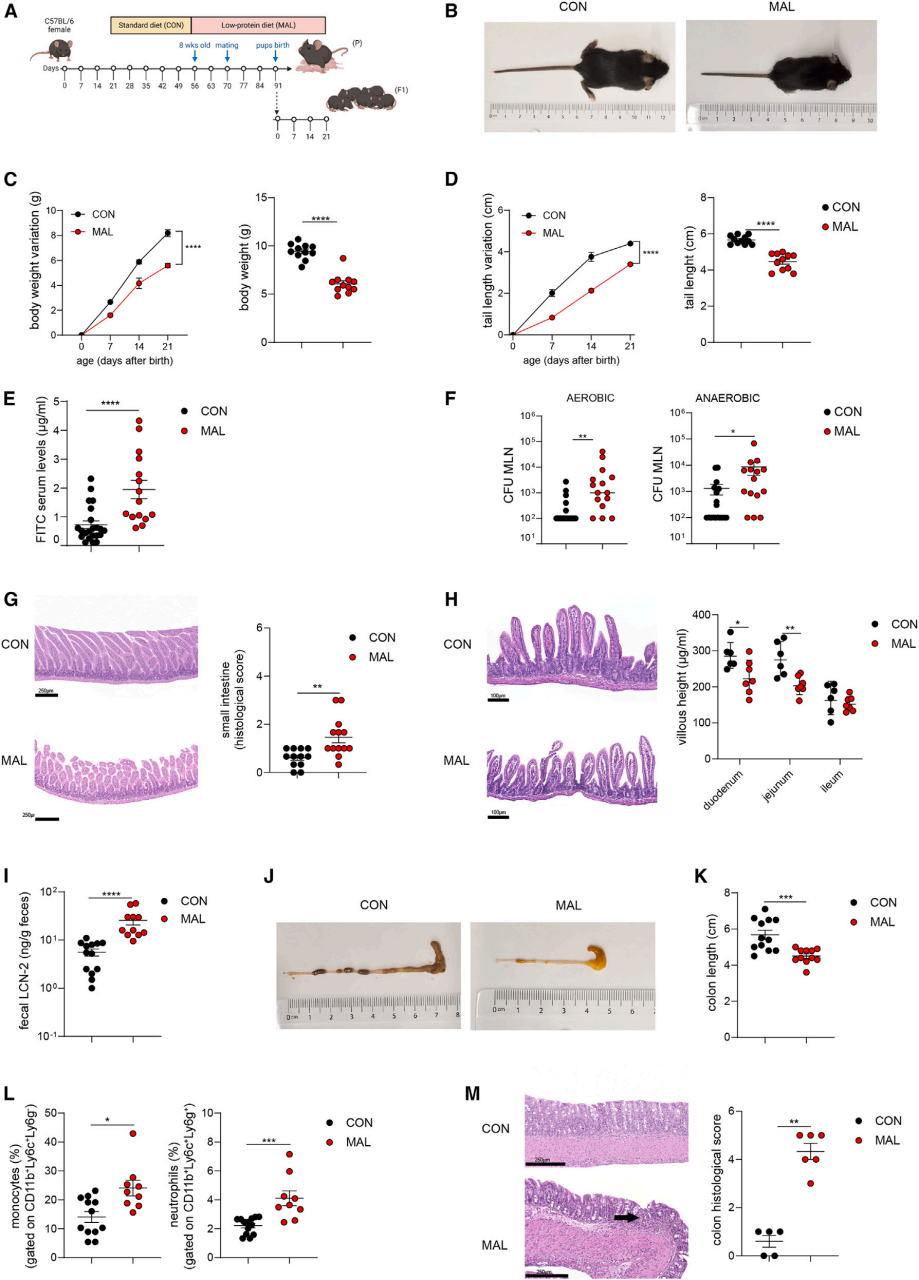
Early-life malnutrition impairs growth and replicates features of environmental enteric dysfunction (EED) Twenty-one-day-old mice were born from CON mothers (black dots) or MAL mothers (red dots). (A) Experimental layout showing the intergenerational MAL model, involving MAL adult C57BL/6 female mice (parental generation, P) and their pups (first filial generation, F1). (B) Representative pictures of CON and MAL pups at weaning. (C) Body weight variation over 21 days after birth (left) and body weight at 21 days after birth (right), (two-way ANOVA, left and Mann-Whitney U test, right). (D) Measurement of tail length variation (cm) over 21 days after birth (left) and tail length at 21 days after birth (right), (two-way ANOVA, left and Mann-Whitney U test, right). (E) Concentration of FITC in the serum was assessed 4 h post oral administration of FITC-dextran, in CON and MAL pups, 21 days after birth (Mann-Whitney U test). (F) Colony-forming unit (CFU) quantification of aerobic and anaerobic bacteria recovered from the MLN (Mann-Whitney U test). (G) Representative H&E sections of the small intestine and statistical analysis of histopathological scores. Scale bar: 250 μm (Mann-Whitney U test). (H) Representative H&E sections of the jejunum villous and statistical analysis of villus height (μm) in the different small intestinal parts (*n* = 7 mice per group, one independent experiment, two-way ANOVA). Scale bar: 100 μm. (I) Fecal LCN-2 concentration (ng/g feces), (Mann-Whitney U test). (J) Representative pictures of large intestine from CON and MAL pups. (K) Colon lengths (cm), (Mann-Whitney U test). (L) Statistical analysis of monocytes (CD45^+^CD11b^+^Ly6c^+^Ly6g^−^, left) and neutrophils (CD45^+^CD11b^+^Ly6c^+^Ly6g^+^, right) infiltrating the colon lamina propria (Mann-Whitney U test). (M) Representative H&E sections of the colon and statistical analysis of histopathological scores. Black arrow: mucosal erosion. Scale bar: 250 μm (*n* = 5–6 mice per group, one independent experiment, Mann-Whitney U test). Data points represent individual mice; unless indicated otherwise, data are pooled from three independent experiments, and statistics are displayed as mean ± SEM. ∗*p* < 0.05; ∗∗*p* < 0.01; ∗∗∗*p* < 0.001; *p* < 0.0001.

Existing mouse models of EED require interventions on both the diet and microbiota to reproduce symptoms of the disease.^22^^,^^23^^,^^24^ To establish a more faithful model of this condition that avoids intestinal colonization with “exogenous bacteria,” we established an intergenerational model of malnutrition, in which prospective dams were fed an impoverished diet. Eight-week-old C57BL/6 female mice were randomized into two groups and fed with either MAL (7% proteins; 5% fat) or conventional (CON: 20% proteins; 15% fat) diet for 2 weeks prior to mating (Figure 1A; Figure S1A). Fifteen days after diet modification, MAL mice did not show significant differences in body weight gain compared to the CON counterpart (Figure S1B); however, the incidence of pregnancy was negatively affected by the MAL diet (Figure S1C). Compared to CON, MAL dams showed impaired gut barrier integrity, as measured by increased permeability to fluorescein isothiocyanate (FITC)-dextran (Figure S1D) and enhanced dissemination of both aerobic and anaerobic bacteria to mesenteric lymph nodes (MLNs) (Figure S1E). MAL dams developed mild signs of colon inflammation, quantified as colon shortening (Figure S1F), and increased fecal lipocalin-2 (LCN-2) levels (Figure S1G), suggesting malnutrition affected both the small intestine and the colon structure. To understand if maternal malnutrition could have an impact on offspring growth and mucosal immune system development, neonates born from CON or MAL dams were daily checked after birth, and their growth rate was assessed until weaning (21–23 days after birth). Weanling mice fed by MAL mothers exhibit signs of growth stunting as evidenced by a reduction of size (Figure 1B), body weight gain (Figure 1C), and tail length (Figure 1D) over time. In humans, EED is characterized by reduced intestinal barrier function, mucosal inflammation with immune cell infiltration, and a reduction in the length of intestinal villi (“blunting”).^25^^,^^26^ Analogously, MAL offspring at weaning exhibited impaired gut barrier integrity as assessed by permeability to FITC-dextran (Figure 1E) and both aerobic and anaerobic bacterial colonization of MLNs (Figure 1F). The histochemical analysis of the small intestine revealed significantly increased histological score values (Figure 1G) and significantly reduced villi length in the duodenum and jejunum (Figure 1H) from MAL as compared to CON pups. Moreover, the MAL offspring was characterized by increased levels of fecal LCN-2 (Figure 1I), shortening of the colon (Figures 1J and 1K), and infiltration of inflammatory monocytes and neutrophils in the colon lamina propria (Figure S2A; Figure 1L) that was associated with epithelial damage and lymphocytic infiltration (Figure 1M). Therefore, a diet low in protein and fat in dams sets the stage for EED-like disease in offspring by promoting intestinal inflammation and limiting growth.

### Reduced SIgA levels and altered adaptive immune system development in MAL weanling mice

Breastfeeding shapes the gut microbiota and immune system development in early life, both directly by exposure of the neonate to the milk microbiota and indirectly, via maternal milk factors that affect bacterial growth and metabolism, such as oligosaccharides, SIgA, and anti-microbial factors.^27^ The analysis of total protein concentration in breast milk collected from MAL dams did not reveal significant differences with respect to CON mice despite the lower protein content of MAL vs. CON diet (Figure S1H). During pregnancy, IgA-expressing plasma cells migrate from the gut to the mammary gland to ensure SIgA release in the breast milk.^28^ Inspection of Peyer’s patches (PPs) in CON and MAL dams at 21 days after delivery revealed significantly reduced cellularity (Figure S1I), with a significant reduction of T follicular helper (Tfh) (Figure S1J; S2B), T follicular regulatory (Tfr) (Figure S1K; S2B), germinal center (GC), albeit not significant, B (Figure S1L; S2C), and IgA-secreting cells (Figure S1M) in MAL with respect to CON mice. Accordingly, SIgA concentration in ileal fluids collected from MAL dams was significantly reduced (Figure S1N). These modifications of gut-associated lymphoid tissue (GALT) function were associated to a significant reduction of SIgA concentration in breast milk from MAL dams (Figure S1O), suggesting that the altered mucosal homeostasis caused by malnourishment in MAL dams could impact the offspring’s intestinal development via the impaired provision of SIgA by breast milk. SIgA reduction in breast milk of MAL dams was associated with a moderate albeit significant increase of IgM (Figure S1P). Even though the increase of IgG levels we detected in the breast milk from MAL vs. CON dams was not statistically significant (Figures S1Q), the analysis of IgG subclasses revealed significant increases of IgG2a, IgG2b, and IgG3 with a slight reduction of IgG1 in MAL dams (Figures S1R). These data suggest that the intestinal alterations observed in MAL dams favor systemic spreading of bacterial antigens that results in enhanced secretion of T cell-independent Igs in the breast milk of MAL dams.^29^ Moreover, they highlight the detrimental effects of malnutrition on dams, ranging from mild effects on the gut adaptive immune system to severe modifications of Ig composition in breast milk.

**Figure 2:**
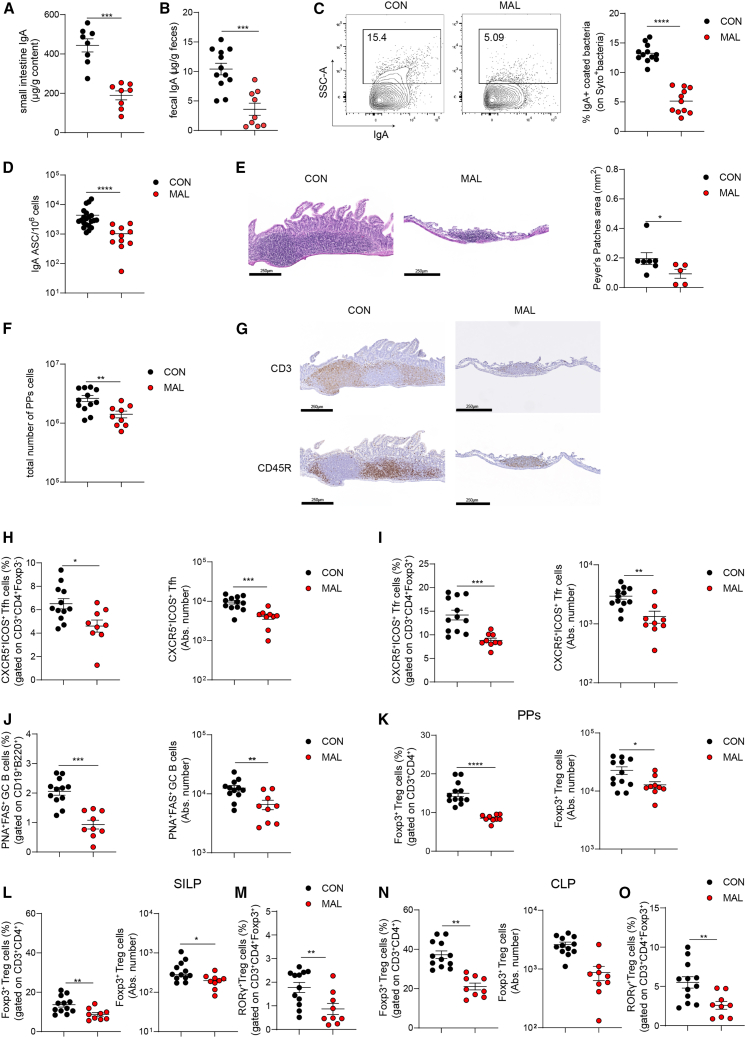
Early-life malnutrition induces an altered development of intestinal adaptive immune system (A and B) Quantification of total IgA by ELISA in the small intestine (A) and feces (B) in 21-day-old CON and MAL pups. (C) Statistical analysis of IgA-coated bacteria performed by flow cytometry in the stools of 21-day-old CON and MAL pups. (D) IgA-secreting plasma cells measured by ELISPOT assay in the lamina propria of the small intestine from CON and MAL 21-day mice. (E) H&E staining of PPs from CON and MAL mice and statistics of PPs area. (F) PPs cellularity expressed as total number of PPs cells in CON and MAL pups. (G) Immunohistochemistry of PPs from CON and MAL pups stained with anti-CD3 and anti-CD45R antibodies. (H–K) Frequencies and absolute numbers of Tfh (H), Tfr (I), GC B (J), and Treg (K) cells in PPs from CON and MAL 21-day-old pups. (L–O) Frequencies and absolute numbers of Foxp3^+^ Treg cells and frequency of Foxp3^+^RORγ^+^ Treg cells in the SILP (L, M) and CLP (N, O) of CON and MAL 21-day-old pups (Mann-Whitney U test). Data points represent individual mice, data are pooled from three independent experiments, and statistics are displayed as mean ± SEM. ∗*p* < 0.05; ∗∗*p* < 0.01; ∗∗∗*p* < 0.001; *p* < 0.0001.

In MAL offspring at 21 days after birth, SIgA concentration was significantly reduced in both the small intestine (Figure 2A) and feces (Figure 2B) as compared to the CON counterpart, and bacterial coating by SIgA was also significantly affected (Figure S2D and Figure 2C). We also detected a significant reduction of IgA-secreting plasma cells in the lamina propria of the small intestine from MAL mice (Figure 2D), suggesting that, in addition to the impaired provision of maternal SIgA, defective production of endogenous SIgA could contribute to the overall SIgA reduction. PPs size (Figure 2E) and cellularity (Figure 2F) were significantly reduced in MAL vs. CON pups. Immunohistochemistry of PPs with anti-CD3 and anti-CD45R antibodies showed the rudimentary structure of PPs in MAL pups as compared to the organized distribution of T and B cells in PPs in CON pups (Figure 2G). Immunophenotyping of PPs revealed a reduction of GCs in MAL pups; Tfh (Figure 2H), Tfr (Figure 2I), and GC B cells (Figure 2J) were all significantly reduced. Moreover, in MAL pups, the frequencies of total Foxp3^+^ Treg cells in PPs (Figure 2K) and total Foxp3^+^ and Foxp3^+^RAR-related orphan nuclear receptor (ROR)γ^+^ T regulatory (Treg) cells in small intestinal lamina propria (SILP) (Figures 2L, 2M; Figure S2E) and colon lamina propria (Figures 2N, 2O; Figure S2E) were all reduced with respect to CON pups. Recently, a double-negative feedback loop was shown to inversely correlate RORγ^+^ Treg cell abundance in the colon and SIgA concentration in breast milk; strain-specific differences in milk SIgA levels and differential microbial coating in offspring would set the transmissible induction of RORγ^+^ Treg cells in early life to ensure intestinal homeostasis.^30^ This anticorrelation was lost in MAL pups, in which RORγ^+^ Treg cells were significantly reduced with respect to CON mice despite significantly reduced SIgA levels. We hypothesize that this discrepancy with the proposed model could be due to the strong perturbation induced by the MAL diet on the distribution of Ig isotypes in breast milk with significant increases of IgM, IgG2a, IgG2b, and IgG3 (Figure S1P-R) that could affect this multigenerational regulatory loop. Nevertheless, the increased transfer of secretory IgM and IgG2a/IgG2b/IgG3 via breast milk might limit gut colonization by enteric pathogens as well as the stimulation of proinflammatory cells in the offspring of MAL dams.^31^

SIgA in breast milk contributes to intestinal barrier function and regulates mucosal colonization by the commensal microbiota, reducing the activation of epithelial pattern recognition receptors and downstream inflammatory signaling.^21^ Consistent with a specific role of SIgA in conditioning the gut ecosystem for newborn’s development, *Igha*^*−/−*^ CON pups showed significantly reduced body weight gain (Figure S3A) and tail length (Figure S3B) compared to wild-type (WT) CON mice. Moreover, we observed significantly increased intestinal permeability (Figure S3C) and colon shortening (Figure S3D) in IgA-deficient as compared to WT CON mice, suggesting that SIgA is required for newborn’s intestinal immune homeostasis and the gut ecosystem’s contribution to the organism’s growth.

### Compromised microbiota structure in weanling mice from MAL dams

**Figure 3:**
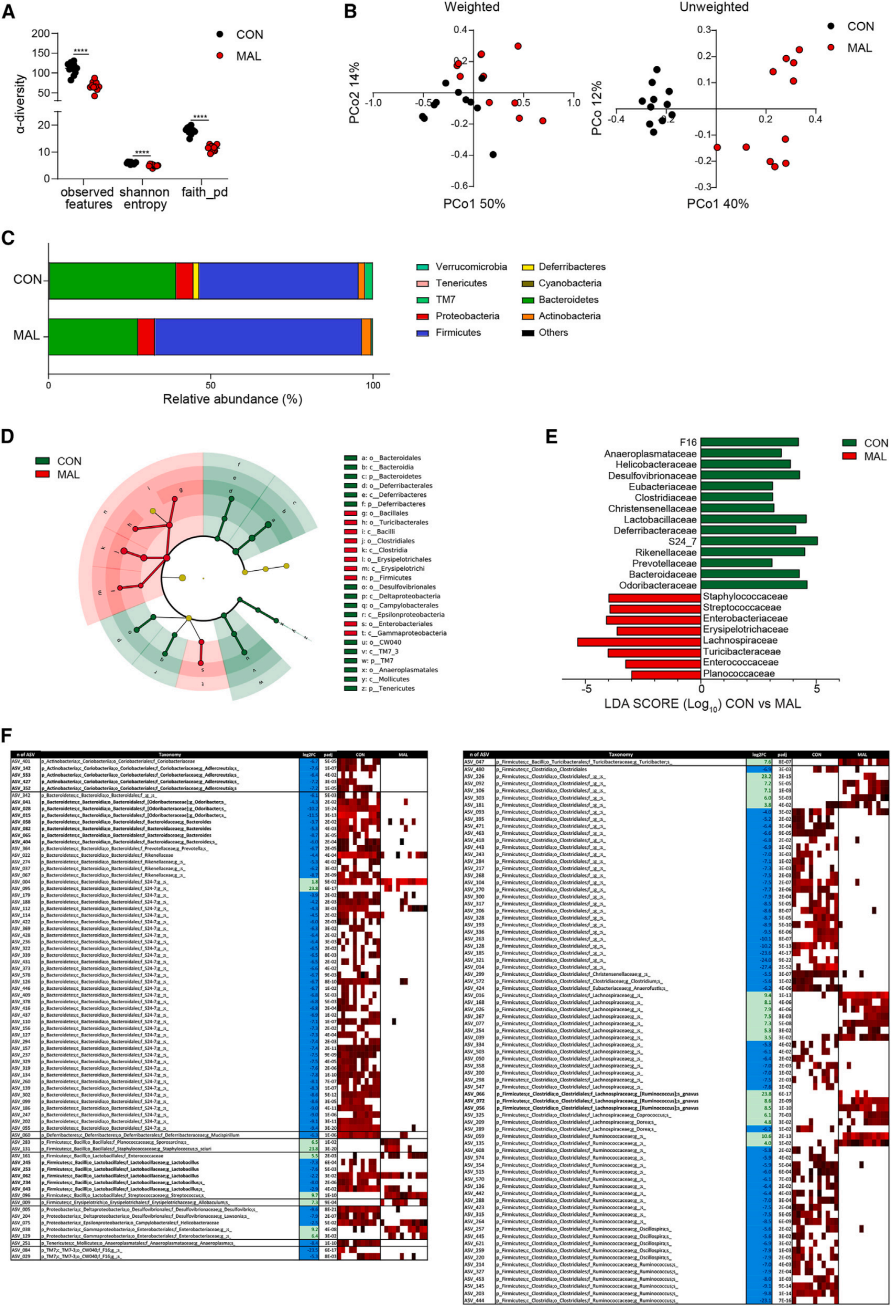
Early-life malnutrition compromises microbiota development (A) Bacterial α-diversity calculated by Shannon index, observed features, and Faith’s phylogenetic diversity. Two-tailed Mann-Whitney U test was used. ∗∗∗∗*p* < 0.0001; data points represent single mice (*n* = 11). (B) Bacterial β-diversity. The PCoA plots of microbial β-diversity were generated using unweighted and weighted UniFrac algorithms. PERMANOVA was used. *p* < 0.001 and *p* < 0.003, respectively. Data points represent single mice (*n* = 11). (C) Phylum relative abundance in CON and MAL 21-day-old mice. (D) Cladogram for orders and (E) linear discriminant analysis (LDA) scores (log10) for the most discriminant bacterial family identified by linear discriminant analysis effect size (LEfSe) in the cecal microbiota in CON and MAL 21-day-old mice. (F) Heatmap showing bacterial amplicon sequence variants (ASVs) in the cecal microbiota that discriminate CON and MAL 21-day-old mice. ASVs were selected according to *p* < 0.05 with Wald test using false discovery rate (FDR) *p* value correction following DESeq2 read counts normalization. Each line represents one ASV, and each column represents an individual mouse. Mean relative abundances (log10) of ASVs detected in the different experimental groups are shown.

To test the impact of dams’ malnutrition on the gut microbiota composition of offspring, we analyzed the bacterial community structure in the caecum of CON and MAL mice by 16S rRNA gene profiling. MAL dams were characterized by a decreased α-diversity (observed features, Shannon index, and Faith’s phylogenetic diversity) of the cecal microbiota (Figure S4A) and clustered apart from CON dams in unweighted and weighted UniFrac analyses (Figure S4B). Analogously, MAL pups showed a strong reduction of bacterial taxonomic richness as reflected by the analysis of α-diversity (Figure 3A). Unweighted and weighted UniFrac analyses visualized on the principal coordinate analysis plot both showed that MAL pups clustered apart from CON mice (permutational multivariate ANOVA [PERMANOVA] unweighted *p* = 0.001; PERMANOVA weighted *p* = 0.003) (Figure 3B). These data suggest that SIgA reduction in breast milk of MAL dams could contribute to the altered development of the microbiota in the offspring.

The analysis of the cecal microbiota in MAL pups at the phylum level revealed marked alterations with respect to CON mice. MAL mice were characterized by a significant enrichment of *Firmicutes* that was counterbalanced by a decrease in *Bacteroidetes*, *Deferribacteres*, *Tenericutes*, *and TM7* phyla (Figures 3C; Table S1)*.* Among *Firmicutes*, MAL mice were characterized by the increase of the orders *Bacillales*, *Turicibacterales*, *Clostridiales*, *Erysipelotrichales*, and *Enterobacteriales* (Figure 3D). In MAL mice, families with a potential etiopathogenic role in inflammation and permeability, including *Staphylococcaceae*, *Streptococcaceae*, *Enterobacteriaceae*,^32^ *Turicibacteriaceae*,^33^ and *Enterococcaceae*,^34^ were significantly enriched with respect to CON mice (Figure 3E). Moreover, MAL mice were characterized by higher relative abundances of *Erysipelotricaceae* and *Lachnospiraceae*, a finding associated with non-breastfeeding in a comprehensive analysis of infant’s microbiome^35^^,^^36^ (Figure 3E). Among the families that were depleted in MAL pups, the reduction of *Clostridiaceae* could be of pathogenetic significance for EED since bacteria belonging to this family were shown to be protective against infections and intestinal damage.^37^ The depletion of *Rikenellaceae* might also be important in EED pathophysiology because of its association with beneficial functions such as early priming of the immune system^38^ (Figure 3E). More in-depth taxonomic analysis (Figure 3F) revealed the depletion of the genus *Adlercreutzia*, which has been associated with anti-inflammatory properties via the production of beneficial metabolites.^39^ We also found a reduction of *Lactobacillus*, a genus which has been shown to be reduced in MAL children^40^; moreover, its provision in immunocompromised-MAL mice improved the resistance against intestinal and respiratory infections,^41^ suggesting it can contribute a beneficial function in malnutrition. Other bacterial genera with possible pathogenetic relevance that were depleted in MAL mice included *Odoribacter* and *Bacteroides*.^32^^,^^42^ Among the species significantly enriched in MAL pups, *Ruminococcus gnavus* has been strongly associated to inflammatory bowel disease (IBD) in a number of studies at different geographical locations.^43^^,^^44^^,^^45^^,^^46^ These data demonstrate that malnourishment in mothers can cause a significant perturbation of the microbiota structure in neonates, which could promote dysbiotic and inflammatory features.

### Administration of apyrase-releasing *Lactococcus lactis* to MAL weanling mice ameliorates stunted growth and enteropathic features

**Figure 4:**
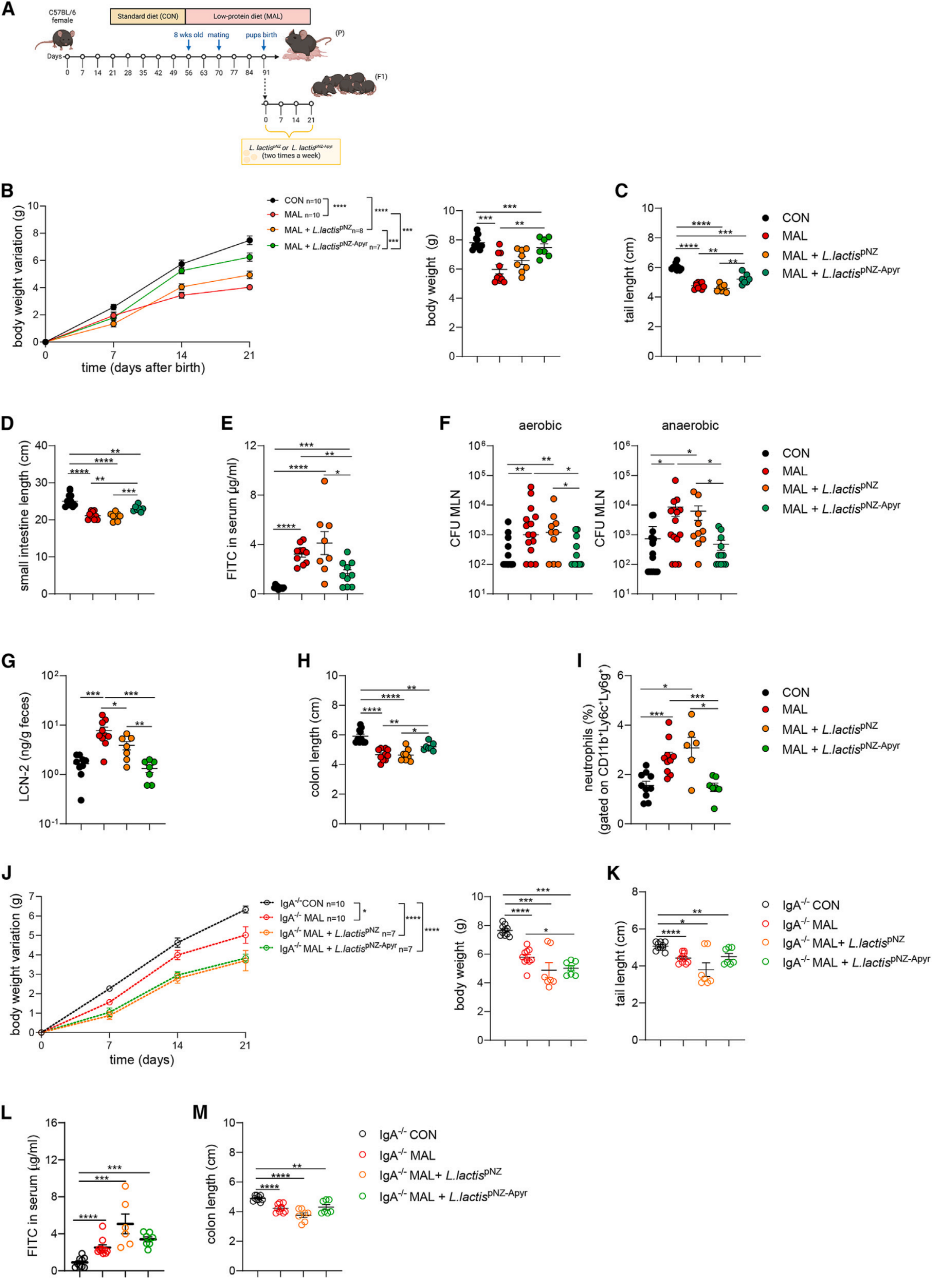
*Lactococcus* *lactis*^pNZ-Apyr^ treatment ameliorates growth and prevents development of EED in MAL newborns Twenty-one-day-old mice were born from CON mothers (black dots) or MAL mothers (red dots); starting from 24 h after birth, pups were gavaged twice a week for 21 days with *L. lactis*^pNZ^ or *L. lactis*^pNZ-Apyr^. (A) Experimental layout showing the intergenerational MAL model, involving MAL adult C57BL/6 female mice (parental generation, P) and their pups (first filial generation, F1). Starting from 24 h after birth, MAL pups were gavaged twice a week with *L. lactis*^pNZ^ or *L. lactis*^pNZ-Apyr^ until day 21. (B) Body weight variation over 21 days after birth (left) and body weight at 21 days after birth (right) (two-way ANOVA, left and Mann-Whitney U test, right). (C and D) Measurement of tail length (C) and small intestine length (D) at 21 days after birth (Mann-Whitney U test). (E) Concentration of FITC in the serum was assessed 4 h post oral administration of FITC-dextran, in CON and MAL pups treated with *L. lactis*^pNZ^ or *L. lactis*^pNZ-Apyr^, 21 days after birth (Mann-Whitney U test). (F) CFUs of aerobic and anaerobic bacteria recovered from the MLN (Mann-Whitney U test). (G) Fecal LCN-2 concentration (ng/g feces) (Mann-Whitney U test). (H) Colon lengths (cm) (Mann-Whitney U test). (I) Statistical analysis of neutrophils (CD45^+^CD11b^+^Ly6c^+^Ly6g^+^) infiltrating the colon lamina propria (Mann-Whitney U test). Data points represent individual mice, data are pooled from three independent experiments, and statistics are displayed as mean ± SEM. The malnutrition model was reproduced in IgA-deficient mice (IgA^−/−^) mice where 21-day-old IgA^−/−^ mice were born from IgA^−/−^ CON mothers (black dots) or IgA^−/−^ MAL mothers (red dots); starting from 24 h after birth, IgA^−/−^ pups were gavaged twice a week for 21 days with *L. lactis*^pNZ^ or *L. lactis*^pNZ-Apyr^. (J) Body weight variation over 21 days after birth (left) and body weight at 21 days after birth (right) (two-way ANOVA, left and Mann-Whitney U test, right). (K) Tail length at 21 days after birth (Mann-Whitney U test). (L) Concentration of FITC in the serum was assessed 4 h post oral administration of FITC-dextran, in 21-day-old CON and MAL pups treated with *L. lactis*^pNZ^ or *L. lactis*^pNZ-Apyr^ (Mann-Whitney U test). (M) Colon length at 21 days after birth (Mann-Whitney U test). Data points represent individual mice, data are pooled from three independent experiments, and statistics are displayed as mean ± SEM. ∗*p* < 0.05; ∗∗*p* < 0.01; ∗∗∗*p* < 0.001; *p* < 0.0001.

We have previously shown that extracellular ATP (eATP) released by the microbiota in the small intestine permeates GALT and limits Tfh cell activity and GC reaction.^47^ The hydrolysis of intestinal eATP by oral gavage of live biotherapeutics engineered to release ATP-diphosphohydrolase (apyrase) from *Shigella flexneri* in the terminal ileum results in greater GC reaction, amplification of the SIgA repertoire with enhanced SIgA coating of microbiota, and significant amelioration of antibiotic-mediated dysbiosis.^48^ We addressed whether this treatment could improve the enteropathic features via SIgA-mediated microbiota adaptation. We selected *Lactococcus lactis* as the probiotic strain to deliver apyrase to the terminal ileum of pups because of its clinical safety^49^ and anti-inflammatory properties^50^ (Figures S5A and S5B). Oral gavage of apyrase-expressing *Lactococcus lactis* (*L. lactis*^pNZ-Apyr^) transformants resulted in effective degradation of intestinal ATP in specific pathogen-free mice (Figure S5C). To understand if apyrase could have an impact on growth parameters in the offspring of MAL dams, neonates were orally gavaged in the first 24 h after birth and then twice a week until day 21 with 10^8^ *L. lactis*^pNZ^ control transformants or *L. lactis*^pNZ-Apyr^ (Figure 4A). Notably, *L. lactis*^pNZ-Apyr^ administration to MAL pups resulted in the significant improvement of growth parameters, including body weight gain over time (Figure 4B), as well as tail (Figure 4C) and small intestine length (Figure 4D) as compared to control and *L. lactis*^pNZ^-treated MAL mice. Moreover, the treatment with *L. lactis*^pNZ-Apyr^ resulted in a significant improvement of intestinal permeability (Figure 4E), a reduction of the translocation of both aerobic and anaerobic bacteria into MLNs (Figure 4F), and amelioration of intestinal inflammation, as demonstrated by lower levels of LCN-2 in the stools (Figure 4G), milder shortening of the colon (Figure 4H), and reduced recruitment of neutrophils into the lamina propria (Figure 4I) as compared to control and *L. lactis*^pNZ^-treated offspring. These data indicate that *L. lactis*^pNZ-Apyr^ supplementation can significantly ameliorate the low-grade intestinal inflammation observed in MAL offspring and the consequences of enteropathy. To understand whether SIgA was involved in apyrase-mediated intestinal adaptation to malnutrition, the same experiment was performed using *Iga*^*−/−*^ mice. Malnutrition resulted in a pronounced faltering of body weight gain and the significant reduction of tail length in *Iga*^*−/−*^ MAL pups as compared to CON *Iga*^*−/−*^ animals (Figures 4J and 4K). Intestinal permeability (Figure 4L) and colon length (Figure 4M) were also significantly affected in MAL *Iga*^*−/−*^ MAL vs. CON pups. However, differently from what was observed in WT mice, *L. lactis*^pNZ-Apyr^ administration to *Iga*^*−/−*^ MAL pups did not result in any significant amelioration of these parameters with respect to both control and *L. lactis*^pNZ^-treated *Iga*^*−/−*^ MAL animals (Figures 4J–4M). These data suggest that apyrase-mediated SIgA amplification is important in conferring resistance to gut barrier impairment induced by malnutrition.

### Apyrase promotes SIgA response and Treg cells expansion in MAL newborns

**Figure 5:**
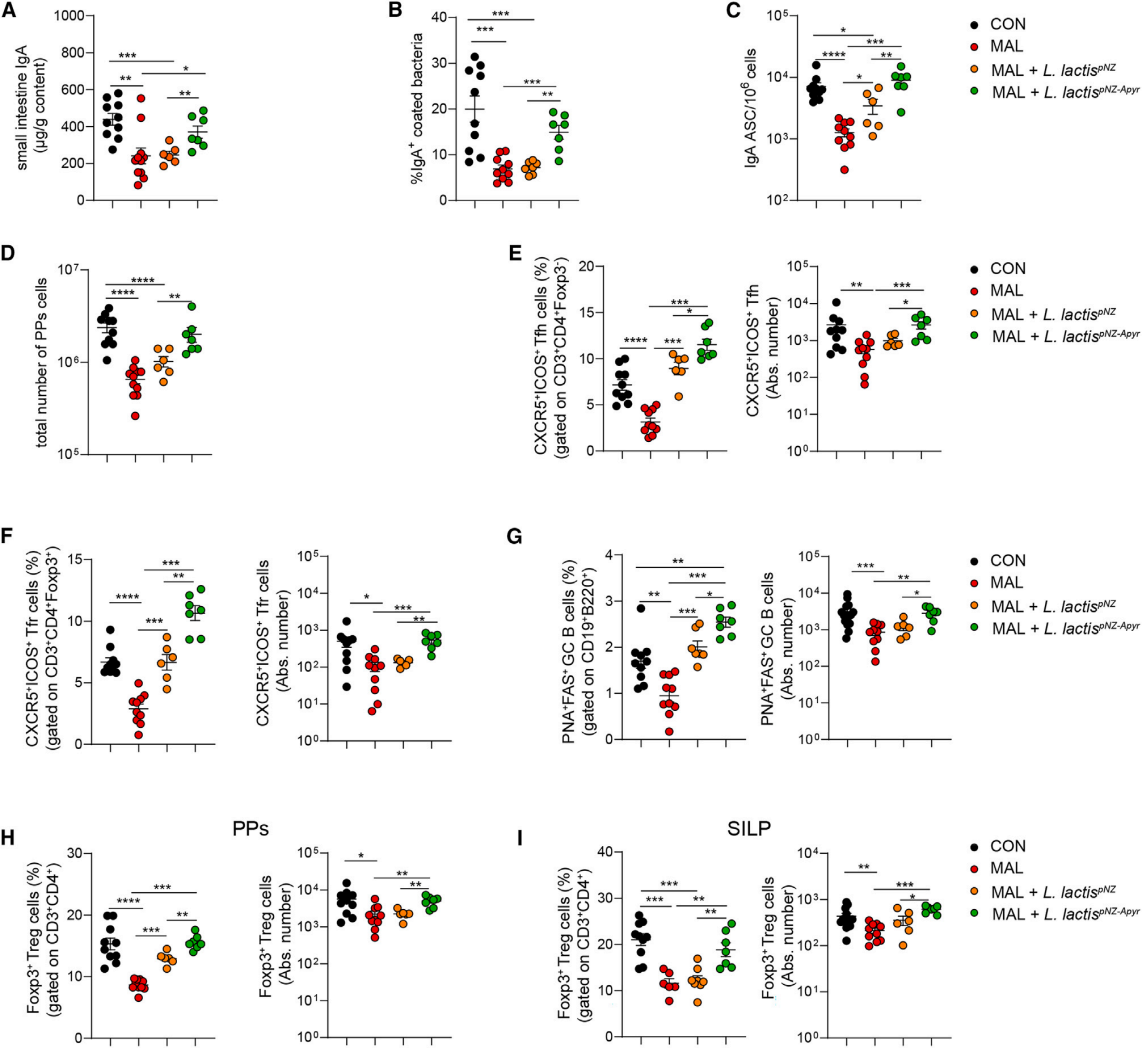
*Lactococcus* *lactis*^pNZ-Apyr^ treatment ameliorates adaptive immune system development in the small intestine of MAL newborns Twenty-one-day-old mice were born from CON mothers (black dots) or MAL mothers (red dots); starting from 24 h after birth, pups were gavaged twice a week for 21 days with *L. lactis*^pNZ^ or *L. lactis*^pNZ-Apyr^. (A) Quantification of total IgA by ELISA in the small intestine of 21-day-old CON and MAL pups. (B) Statistical analysis of IgA-coated bacteria performed by flow cytometry in the stools of 21-day-old CON and MAL pups treated with *L. lactis*^pNZ^ or *L. lactis*^pNZ-Apyr^. (C) IgA-secreting plasma cells measured by ELISPOT assay in the lamina propria of the small intestine from CON and MAL 21-day-old mice treated with *L. lactis*^pNZ^ or *L. lactis*^pNZ-Apyr^. (D) PPs cellularity expressed as total number of PPs cells in CON and MAL pups treated with *L. lactis*^pNZ^ or *L. lactis*^pNZ-Apyr^. (E–G) Frequency of Tfh (E), Tfr (F), and GC B (G) cells in PPs from CON and MAL 21-day-old pups treated with *L. lactis*^pNZ^ or *L. lactis*^pNZ-Apyr^. (H and I) Statistical analysis by flow cytometry of Treg cells in the PPs (H) and SILP (I) of CON and MAL 21-day-old pups treated with *L. lactis*^pNZ^ or *L. lactis*^pNZ-Apyr^ (Mann-Whitney U test). Data points represent individual mice, data are pooled from three independent experiments, and statistics are displayed as mean ± SEM. ∗*p* < 0.05; ∗∗*p* < 0.01; ∗∗∗*p* < 0.001; *p* < 0.0001

We then investigated the effects of apyrase administration during breastfeeding on the development of GCs and SIgA response in MAL pups at weaning. Whereas administration of *L. lactis*^pNZ^ transformants did not produce variations in the concentration of SIgA and percentage of SIgA-coated bacteria in the small intestine with respect to untreated MAL newborns, the treatment with *L. lactis*^pNZ-Apyr^ was sufficient to restore levels of SIgA in MAL pups comparable to those in CON animals (Figure 5A) and to significantly increase the percentage of IgA-coated bacteria with respect to untreated and *L. lactis*^pNZ^-treated animals (Figure 5B). *L. lactis*^pNZ-Apyr^ administration restored the number of IgA-secreting plasma cells in the SILP of MAL pups to levels found in CON mice (Figure 5C). Notably, oral gavaging of *L. lactis*^pNZ^ resulted in the significant increase of IgA-secreting plasma cells as compared to untreated MAL mice (Figure 5C), suggesting *L. lactis* per se could have a beneficial function on the development of a SIgA response in MAL pups. PPs in MAL pups supplemented with *L. lactis*^pNZ-Apyr^ were significantly expanded with respect to untreated and *L. lactis*^pNZ^-treated MAL animals (Figure 5D). As observed with IgA-secreting plasma cells, MAL pups treated with *L. lactis*^pNZ^ showed a significant increase of Tfh, Tfr, and GC B cells with respect to untreated MAL newborns, suggesting that *L. lactis* could promote T cell-dependent SIgA in PPs (Figures 5E–5G). Nevertheless, the provision of apyrase to MAL pups by *L. lactis*^pNZ-Apyr^ resulted in a significant increase of Tfh and Tfr cells with respect to *L. lactis*^pNZ^-treated mice, reaching values similar to those of CON mice (Figures 5E and 5F). The percentage of GC B cells recovered from PPs of pups treated with *L. lactis*^pNZ-Apyr^ was even significantly increased with respect to CON mice (Figure 5G), consistent with the prominent activity of apyrase in fostering the GC reaction in PPs.^51^ Treg cells are particularly sensitive to the induction of cell death by eATP via the P2X7R^52^ and were significantly reduced in the PPs of MAL pups. Albeit *L. lactis*^pNZ^ administration resulted in a significant increase of Treg cells in PPs of MAL mice, *L. lactis*^pNZ-Apyr^ restored Treg cell frequencies to the levels of CON mice (Figure 5H). Notably, whereas Treg cell percentages in SILP of *L. lactis*^pNZ^-treated animals were not different from untreated MAL mice, *L. lactis*^pNZ-Apyr^ administration restored the Treg cell population to levels observed in CON pups (Figure 5I), suggesting apyrase could be particularly effective in limiting the negative effect of eATP on Treg cells in this tissue niche.

### Improvement of gut microbiota in MAL offspring by apyrase

**Figure 6:**
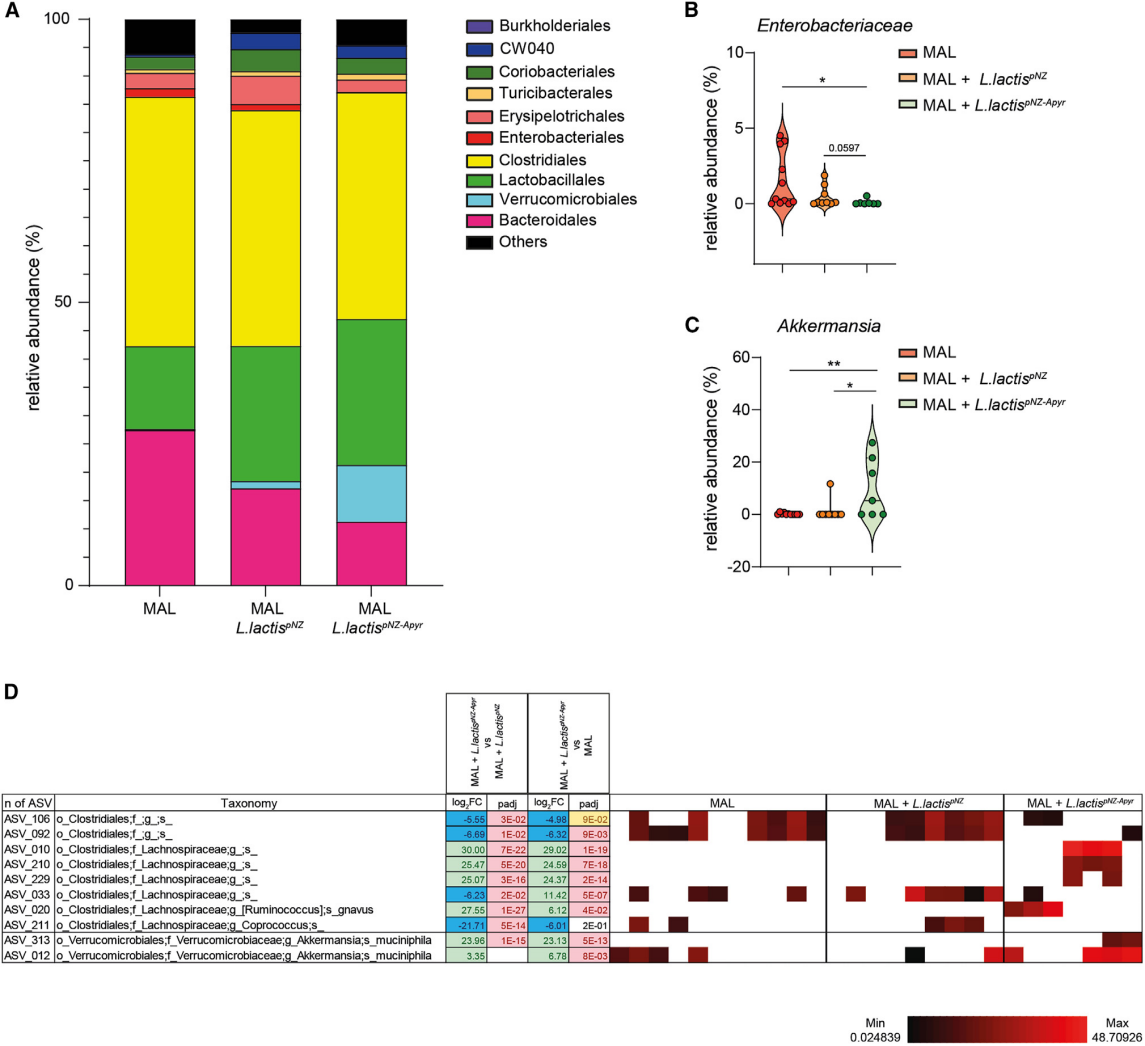
*Lactococcus* *lactis*^pNZ-Apyr^ administration improves gut microbiota selection in MAL newborns (A) Chart summarizing the percent relative abundance of the cecal microbiota by order classification in the different experimental groups (*n* = 7–11 mice per group, one independent experiment). (B and C) Statistical analysis of the relative abundance of *Enterobacteriaceae* (B) and *Akkermansia* (C) in the cecal microbiota in MAL or MAL mice treated with *L. lactis*^pNZ^ or *L. lactis*^pNZ-Apyr^ (*n* = 7–11 mice per group, one independent experiment, Mann-Whitney U test, data points represent individual mice, Mean ± SEM are shown, ∗*p* < 0.05; ∗∗*p* < 0.01). (D) Heatmap showing bacterial ASVs in the cecal microbiota that discriminate 21-day-old MAL or MAL mice treated with *L. lactis*^pNZ^ or *L. lactis*^pNZ-Apyr^. ASVs were selected according to *p* < 0.05 with Wald test using FDR *p* value correction following DESeq2 read counts normalization. Each line represents one ASV, and each column represents an individual mouse. Mean relative abundances (log10) of ASVs detected in the different experimental groups are shown.

The intestinal SIgA response co-evolves with the microbiota from birth and has a fundamental role in modulating microbiota function and ensuring gut homeostasis.^53^ To investigate whether SIgA amplification by apyrase-expressing *L. lactis* could beneficially affect microbial homeostasis in the intestine of MAL offspring, we analyzed by 16S rRNA gene profiling the bacterial community structure in the caecum of MAL pups that were either untreated or orally gavaged with *L. lactis*^pNZ^ and *L. lactis*^pNZ-Apyr^. Unweighted and weighted UniFrac analyses did not reveal any clustering of the differentially treated animals (Figure S6). However, we observed differences at the order and family levels that could contribute to the observed improvement in intestinal function. Administration of .*L. lactis*^pNZ-Apyr^ resulted in the enrichment of *Verrucomicrobiales* and decrease of *Enterobacteriales* in MAL pups as compared to untreated and *L. lactis*^pNZ^-conditioned MAL pups (Figure 6A). A decrease in *Enterobacteriaceae* with respect to MAL pups, albeit not reaching statistical significance, was also observed in mice gavaged with *L. lactis*^pNZ^, suggesting that intestinal colonization by *L. lactis* could synergize with apyrase in depleting this bacterial family (Figure 6B). The relative abundance analysis of differentially represented amplicon sequence variants (ASVs) from the various experimental groups revealed the effect of apyrase in the selective preservation or depletion of bacterial species with potential functional significance in improving the MAL gut ecosystem. The significant increase of *Akkermansia muciniphila* was of particular relevance in this respect (Figures 6C and 6D). Among the *Lachnospiraceae* family, an ASV corresponding to *R*. *gnavus* was enriched and another corresponding to *Coprococcus* was depleted in *L. lactis*^pNZ-Apyr^-treated MAL pups (Figure 6D). Overall, these data reveal a selective effect of apyrase-mediated SIgA amplification in limiting *Enterobacteriaceae* expansion and fostering mucosal colonization by *A*. *muciniphila* in MAL newborns.

### Early intestinal conditioning of MAL newborns with apyrase improves SIgA response to oral immunization with cholera toxin in adulthood

**Figure 7:**
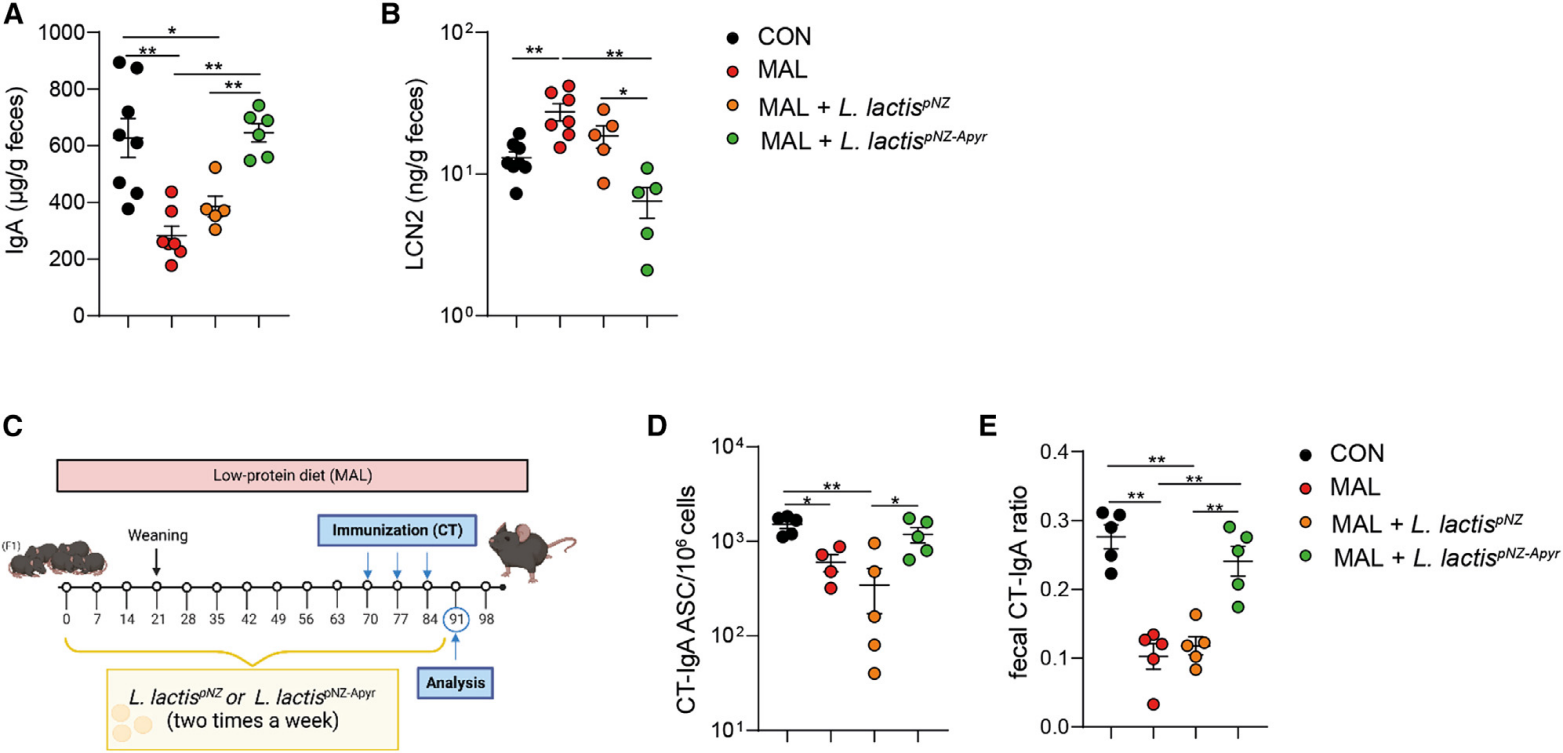
*Lactococcus* *lactis*^pNZ-Apyr^ administration ensures a proficient IgA production in adulthood and improves IgA response to oral CT immunization in MAL mice (A) Quantification of total fecal IgA by ELISA in 10-week-old CON mice, untreated MAL mice, and MAL mice treated with *L. lactis*^pNZ^ or *L. lactis*^pNZ-Apyr^. (B) Fecal LCN-2 concentration (ng/g feces), (Mann-Whitney U test). Data points represent individual mice, and statistics are displayed as mean ± SEM. (C) Experimental design. After weaning, untreated MAL mice or MAL mice treated with *L. lactis*^pNZ^ or *L. lactis*^pNZ-Apyr^ were maintained in low-protein diet and were gavaged with *L. Lactis*^pNZ^ or *L. lactis*^pNZ-Apyr^ twice a week for the entire duration of the experiment. At 10 weeks of age, CON mice and untreated MAL mice or MAL mice treated with *L. lactis*^pNZ^ or *L. lactis*^pNZ-Apyr^ were orally immunized with CT at day 70, 77, and 84. At day 91, mice were analyzed. (D) CT-specific IgA-secreting plasma cells measured by ELISPOT assay in the lamina propria of the small intestine from CON mice, untreated MAL mice, and MAL mice treated with *L. lactis*^pNZ^ or *L. lactis*^pNZ-Apyr^. (E) Quantification of CT-specific IgA to total IgA ratio in feces of CON mice, untreated MAL mice, and MAL mice treated with *L. lactis*^pNZ^ or *L. lactis*^pNZ-Apyr^ (Mann-Whitney U test). Data points represent individual mice, and statistics are displayed as mean ± SEM. ∗*p* < 0.05; ∗∗*p* < 0.01.

EED is considered an important factor in determining the defective responsiveness of affected children to oral vaccinations, which are particularly needed in these subjects due to the overwhelming exposure to enteric pathogens.^54^ We addressed whether the improvement of intestinal homeostasis induced by conditioning MAL pups with *L. lactis*^pNZ-Apyr^ could enhance the responsiveness to oral vaccination with cholera toxin (CT) B subunit, which induces high-titer vaccine-specific SIgA response in the gastrointestinal tract.^55^ In mice colonized with the microbiota from stunted MAL children, the defective gut mucosal IgA response to oral CT vaccination could be corrected by combined prebiotic and microbial intervention.^56^ The analysis of MAL mice at 10 weeks of age before immunization with CT B subunit showed significantly reduced concentrations of IgA and increased levels of LCN-2 in the stool (Figures 7A and 7B). Notably, early intestinal conditioning with *L. lactis*^pNZ-Apyr^, but not *L. lactis*^pNZ^, resulted in restoring these two parameters to the normal values observed in CON mice, indicating that apyrase administration can promote a protracted enhancement of SIgA production and amelioration of the intestinal inflammation that characterizes MAL pups later in life (Figures 7A and 7B). Importantly, *L. lactis*^pNZ-Apyr^ administration prior to oral CT immunization (Figure 7C) resulted in a significant increase of CT-specific IgA plasma cells in the SILP, with respect to untreated and *L. lactis*^pNZ^-treated MAL mice, reaching values similar to CON mice (Figure 7D). Moreover, the ratio of fecal CT-specific IgA to fecal total IgA (referred to as CT-IgA ratio) was increased in *L. lactis*^pNZ-Apyr^ MAL mice compared to *L. lactis*^pNZ^-treated and untreated MAL mice (Figure 7E). Together, these data indicate that apyrase supplementation could restore the response to oral vaccines in MAL conditions.

## Discussion

The adaptation of the intestine to colonization by bacteria at birth is conditioned by a series of interactions involving maternal as well as endogenous factors. Malnutrition and poor sanitary conditions dramatically affect the development of the gut ecosystem. Seminal studies have shown that maturation of the intestinal microbiota structure is disrupted in MAL children and immaturity persists in spite of therapeutic and food interventions, thereby constituting an insidious threat for the organism.^57^^,^^58^ EED is a consequence of malnourishment in children living in LMICs. Stunting and other long-term pathologies or even death can follow on. Early phases of EED are difficult to study in humans; we developed an intergenerational model of malnutrition, which allows investigating how malnutrition affects the imprinting of the mucosal immune system starting from intrauterine life. The offspring from MAL mothers reproduced pathological features of EED, including intestinal inflammation, microbiota alterations, and stunting. An important aspect of this model was the depletion of maternal SIgA in MAL dams. Bacterial coating by passive transfer of SIgA together with nutrients present in breast milk shapes the offspring microbiota structure after birth. MAL pups fed SIgA-depleted breast milk were also characterized by a defect in the generation of endogenous SIgA and other features of the mucosal adaptive immune system, suggesting that maternal SIgA deficiency could have a determinant function in the establishment of EED.

Microbiota-derived eATP in the terminal ileum limits Tfh cell activity and GC reaction in GALT, thereby controlling T cell-dependent SIgA.^47^ The intestinal SIgA repertoire can be amplified by provision of apyrase-releasing live biotherapeutics.^48^ Therefore, to investigate whether this approach could be used to enhance endogenous SIgA generation and ameliorate EED, we gavaged MAL pups with *L. lactis*^pNZ-Apyr^. The administration of *L. lactis*^pNZ-Apyr^, but not control *L. lactis*^pNZ^ transformants, proved to be successful in restoring luminal SIgA concentration and bacterial coating to levels undistinguishable from CON pups. Moreover, it significantly improved growth and ameliorated intestinal inflammation. SIgA was instrumental in determining these outcomes since *Iga*^*−/−*^ MAL pups were not responsive to *L. lactis*^pNZ-Apyr^. The intestinal microbiota of MAL pups was characterized by severe alterations in the relative abundances of various taxa. A prominent effect of the treatment with *L. lactis*^pNZ-Apyr^ was the reduction of *Enterobacteriaceae*, which have been associated to a number of intestinal inflammatory conditions^59^ and disruption of gut barrier integrity when transferred to germ-free mice fed an MAL diet.^23^ This result was consistent with the effective generation of high-affinity SIgA and clearance of *Salmonella* colonizing the intestine by apyrase-mediated SIgA enhancement.^51^ Notably, SIgA-mediated neutralization of *Enterobacteriaceae* pathogenicity was observed in preterm infants at risk of developing necrotizing enterocolitis (NEC), a severe condition in which formula feeding, dysbiosis, and inflammation have all been pathogenically implicated; in these neonates, coating of *Enterobacteriaceae* by maternal SIgA was associated to protection from NEC.^60^

In MAL pups, we observed a significant increase of several *R. gnavus* ASVs. This microbe is a predominant species in infant gut and persists throughout adulthood. This persistence is likely due to the capacity to adapt to human milk oligosaccharide utilization and mucin glycan foraging. Uncapping of mucin glycan chains through its enzymatic machinery not only releases sugars for its subsistence but also provides access to underlying mucin glycan chains to other mucus-colonizing bacteria, such as *A. muciniphila*; therefore, *R. gnavus* plays a pivotal role in controlling this nutritional niche.^61^ Microbiota dysfunction during intestinal inflammation was shown to result in the shift from the health-associated bacterial composition to the disproportionate increase of *R. gnavus* and severe reduction of *A. muciniphila*.^46^ The increased oxidative stress characterizing dysbiosis in IBD led to the blooming of an *R. gnavus* clade bearing genes involved in oxidative stress responses, adhesion, iron acquisition, and mucus utilization, which likely conferred a competitive advantage to *R. gnavus* and perpetuated inflammation.^43^ Conversely, an *R. gnavus* strain ameliorated the impaired growth phenotype of germ-free mice gavaged with an undernourished donor’s immature microbiota, suggesting it could beneficially condition the host metabolic machinery.^62^ *R. gnavus* strains can have differential effects on intestinal homeostasis. In fact, a capsular polysaccharide present in some strains covers the proinflammatory layers of the bacterial cell wall and promotes immunosuppression, whereas unencapsulated strains stimulate a proinflammatory immune response.^63^ Moreover, the capacity of *R. gnavus* strains to elicit anti- or proinflammatory responses is also dependent on the extent of cell wall processing by lysozyme produced by Paneth cells. When gut barrier is compromised, lysozyme-processed *R. gnavus* may worsen inflammation.^64^ Rebuilding of intestinal colonization by *A. muciniphila* in MAL pups treated with *L. lactis*^pNZ-Apyr^ suggests SIgA amplification may contribute its beneficial influence on systemic metabolism also by promoting a physiological colonization of the mucus niche.

The reduction of SIgA-coated bacteria in the small intestine of MAL pups was associated to the significant depletion of the local resident genus *Lactobacillus*, a target of SIgA. Restoring SIgA coating of the microbiota by *L. lactis*^pNZ-Apyr^ administration did not modify the relative abundance of *Lactobacillus*, which was however enriched by Tfh cell-mediated SIgA amplification.^48^ Interestingly, SIgA binding to *Lactobacillus* is conditioned by diet, and isolates from undernourished animals are characterized by the loss of IgA-binding ability due to bacterial adaptation to nutritional pressure.^65^ It would be interesting to see whether applying a normal diet in the presence of apyrase to MAL mice would result in enhancing SIgA coating of *Lactobacillus*. Whatever the outcome would be, our experiments show that SIgA amplification can improve growth and intestinal homeostasis with relatively small modifications of small intestinal microbiota composition, suggesting this beneficial effect is the result of SIgA-mediated reshaping of the biogeography and functional adaptation of the MAL microbiota. In this respect, it is worth considering the aberrant systemic exposure to commensal microbes and immune dysregulation in pediatric subjects lacking intestinal SIgA.^66^ We hypothesize that therapeutic strategies leveraging the dominant function of endogenous SIgA in intestinal homeostasis may prove effective in correcting dysbiosis and its consequences on host organisms, irrespective of the actual microbial ecology.

### Limitations of the study

Since eATP has pleiotropic effects on different cells populating the intestinal environment, it is possible that the observed increase of IgA-secreting cells in the lamina propria of pups treated with apyrase-releasing *L. lactis* could in part benefit from the effect of luminal eATP degradation on other cells. The lack of effect of apyrase-releasing *L. lactis* in improving EED phenotype in IgA-deficient mice supports the function of the enzyme on SIgA amplification as the mechanism responsible for the therapeutic effect. However, we cannot rule out additional beneficial effects of apyrase nor define how apyrase-mediated improvement of intestinal homeostasis results in a proficient CT-specific IgA response. In order to provide an isocaloric diet to MAL mice, the MAL diet was characterized by an increased content of sugar, a characteristic not reflecting the “real-life” situation of malnutrition and that can affect gut permeability and susceptibility to inflammation. We could not determine to which extent this effect contributed to the observed phenotype in MAL mice. Whenever possible, we used littermates that were separated to generate the two groups of CON and MAL prospective dams. Therefore, at the beginning of the experiment, the animals were characterized by similar microbiota. However, our data do not allow dissecting the respective function of MAL diet and microbiota composition in determining the offspring phenotype. Finally, the use of cecal contents for the taxonomic analysis of the microbiota gives a limited description of the intestinal microbial community.

## STAR★Methods

### Key resources table

**Table:** 

REAGENT or RESOURCE	SOURCE	IDENTIFIER
**Antibodies**		

Anti-mouse CD3ε (145-2C11) APC/Cy7	BioLegend	Cat.#:100330; RRID:AB_1877170
Anti-mouse CD4 (GK1.5) PE/Cy7	BioLegend	Cat.#:100422; RRID:AB_312706
Anti-mouse CD25 (PC61) PE	BioLegend	Cat.#:102007; RRID:AB_312857
Anti-mouse ICOS (C398.4A) PerCP/Cy5.5	BioLegend	Cat.#:313518; RRID:AB_10641280
Anti-mouse CD8α (53-6.7) PB	BioLegend	Cat.#:100725; RRID:AB_493426
Anti-mouse CD45 (30-F11) FITC	BioLegend	Cat.#:103108; RRID:AB_312972
Anti-mouse FOXP3 (FJK-16s) APC	eBioscience	Cat.#:17-5773-82; RRID:AB_469457
Anti-mouse B220 (RA3-6B2) PB	BioLegend	Cat.#:103227; RRID:AB_492877
Anti-mouse CD45 (30-F11) PE	BioLegend	Cat.#:103106; RRID:AB_312971
Anti-mouse CD19 (145-2C11) APC/Cy7	BioLegend	Cat.#:115530; RRID:AB_830707
Anti-mouse IgA FITC	Southern Biotech	Cat.#:1040-02; RRID:AB_2794370
Anti-mouse IgG (Poly4053) PE/Cy7	BioLegend	Cat.#:45315
Anti-mouse TCRβ (H57-597) FITC	BioLegend	Cat.#:109212; RRID:AB_313435
Anti-mouse PNA (FL-107) FITC	Vector Laboratories, Inc.	Cat.#:FL-1071; RRID:AB_2315097
Anti-mouse FAS (Jo2) PE	BD Biosciences	Cat.#:554258; RRID:AB_395330
Anti-mouse TCRβ (H57-597) APC	BioLegend	Cat.#:109212; RRID:AB_313435
Anti-mouse CD4 (RM4-5) APC/Cy7	BioLegend	Cat.#:100526; RRID:AB_312726
Anti-mouse CD25 (PC61) PE/Cy7	BioLegend	Cat.#:102016; RRID:AB_312864
Anti-mouse ICOS (15P9) PE	BioLegend	Cat.#:107706; RRID:AB_313335
Anti-mouse CXCR5 (2G8) Biotinylated	BD	Cat.#:551960; RRID:AB_394301
Anti-mouse PD1 (RMP1-30) PerCP/Cy5.5	BioLegend	Cat.#:109120; RRID:AB_2566641
Anti-mouse FOXP3 (FJK-16s) FITC	eBioscience	Cat.#:11-5773-82; RRID:AB_465243
Anti-mouse CD11b (M1/70) PE/Cy7	BioLegend	Cat.#:101216; RRID:AB_312799
Anti-mouse Ly6G (1A8) PB	BioLegend	Cat.#:127612; RRID:AB_1877212
Anti-mouse Ly6C (HK1.4) Biotinylated	BioLegend	Cat.#:128004; RRID:AB_1236553
Anti-human/mouse RORγ(t) (FKJS-9) PE	eBioscience	Cat.#:12-6988-82; RRID:AB_1834470
Anti-mouse CD45R/B220 (RA3-6B2), Purified	BD	Cat.#:550286; RRID:AB_394614
Anti-CD3 epsilon antibody (SP7)	Abcam	Cat#:ab16669; RRID:AB_443425
Rabbit anti-mouse IgA (mA-6E1) APC conjugated	Invitrogen	Cat.#:17-4204-82; RRID:AB_2848294
Goat anti-mouse IgA unlabeled	Southern Biotechnologies	Cat.#:1040-01; RRID:AB_2314669
Goat anti-mouse IgG unlabeled	Southern Biotechnologies	Cat.#:1031-01; RRID:AB_2794303
Goat anti-mouse IgG1 unlabeled	Southern Biotechnologies	Cat.#:1070-01; RRID:AB_2794408
Goat anti-mouse IgG2c unlabeled	Southern Biotechnologies	Cat.#:1079-01; RRID:AB_2794464
Goat anti-mouse IgG2b unlabeled	Southern Biotechnologies	Cat.#:1090-01; RRID:AB_2794517
Goat anti-mouse IgG3 unlabeled	Southern Biotechnologies	Cat.#:1100-01; RRID:AB_2794567
Goat anti-mouse IgM unlabeled	Southern Biotechnologies	Cat.#:1021-01; RRID:AB_2687524
Goat anti-mouse IgA alkaline phosphatase (AP) conjugated	Southern Biotech	Cat.#:1040-04; RRID:AB_2794372
Goat anti-mouse IgG alkaline phosphatase (AP) conjugated	Southern Biotech	Cat.#:1030-04; RRID:AB_2794293
Goat anti-mouse IgG1 alkaline phosphatase (AP) conjugated	Southern Biotech	Cat.#:1070-04; RRID:AB_2794411
Goat anti-mouse IgG2c alkaline phosphatase (AP) conjugated	Southern Biotech	Cat.#:1079-04; RRID:AB_2692321
Goat anti-mouse IgG2b alkaline phosphatase (AP) conjugated	Southern Biotech	Cat.#:1090-04; RRID:AB_2794520
Goat anti-mouse IgG3 alkaline phosphatase (AP) conjugated	Southern Biotech	Cat.#:1100-04; RRID:AB_2794572
Goat anti-mouse IgM alkaline phosphatase (AP) conjugated	Southern Biotech	Cat.#:1021-04; RRID:AB_2794239
Mouse IgA unlabeled	Southern Biotech	Cat.#:0106-01; RRID:AB_2714214
Anti-IgA biotin conjugated	Southern Biotech	Cat.#:1040-08; RRID:AB_2794374

**Bacterial and virus strains**		

*L.lactis*^*PNZ*^	In this paper	N/A
*L.lactis*^*PNZ-Apyr*^	In this paper	N/A

**Chemicals, peptides, and recombinant proteins**		

Streptavidin PercP/Cy5.5	BioLegend	Cat.#:405213
Streptavidin eF450	eBioscience	Cat.#:48-4317-82
Streptavidin horseradish peroxidase conjugate (HRP)	Invitrogen	Cat.#:S911
FITC-Dextran (MW4000)	Sigma Aldrich	Cat.#:D8537
HEPES	Gibco	Cat.#:15630-056
M17 medium	Merk	Cat.#:CM0817B
Glucose	Sigma Aldrich	Cat.#:G7021
Nisin	Sigma Aldrich	Cat.#:N5764
Schaedler agar	Sigma Aldrich	Cat.#:91019
Bovine Serum Albumine	Sigma Aldrich	Cat.#:A7906
RPMI Medium 1640(1x)	Gibco	Cat.#:42401-018
Penicillin/streptomycin	Gibco	Cat.#:15070-063
Kanamycin	Gibco	Cat.#:15160-047
EDTA - solution pH 8,0	Axon Lab	Cat.#:A3145.1000
0.05% Trypsin-EDTA	Gibco	Cat.#.25300-054
Collagenase D from Clostridium histolyticum	Sigma Aldrich	Cat.#:11088882001
DNAse I grade II	Sigma Aldrich	Cat.#:10104159001
Fetal bovine serum	Gibco	Cat.#:10270-106
Percoll	Cytiva	Cat.#:17089101
Dulbecco’s Phosphate Buffered Saline (PBS)	Sigma-Aldrich	Cat.#:D8537
SYTO™ BC Green Fluorescent Nucleic Acid Stain	Life Technologies	Cat.#:S34855
Goat serum	Sigma	Cat.#:G9023
Parafolmadehyde	Merk	Cat.#:30525-89-4
Cholera Toxin antigen	Sigma-Aldrich	Cat.#:C8052
4-Nitrophenyl Phosphate disodium sal hexahydrate	Sigma-Aldrich	Cat.#:N2765-100TAB
Na_2_CO_3_/L	Merk	Cat.#:1.06395.0500
NaCO_3_/L	Applichem	Cat.#:A0384.0500
Souybean trypsin inhibitor	Sigma-Aldrich	Cat.#:T6522
Phenylmethanesylfonyl fluoride (PMSF)	Sigma-Aldrich	Cat.#:P7625
Cholera toxin (CT)	Sigma-Aldrich	Cat.#:C8052
Chicken ovalbumin	Sigma-Aldrich	Cat.#:A5503

**Critical commercial assays**		

Comassie PlusTM Protein assay	Thermo Fisher	Cat.#:23236
DuoSet ELISA Mouse Lipocalin-2/NGAL	R&D Systems	Cat.#:DY1857
ATP Determination Kit	Invitrogen	Cat.#:A22066
Fast DNA Stool Mini Kit	Quiagen	Cat.#51604
Fixation/Permeabilization Solution Kit	BD Bioscience	Cat.#557414
Foxp3/Transcription Factor Staining Buffer Set	eBioscience	Cat.#00-5523-00
Quant-iT™ PicoGreen™ dsDNA Assay Kits	Thermo Fisher	Cat.#P7589
Mouse Lipocalin-2/NGAL DuoSet ELISA	R&D Systems	Cat.#:DY1857

**Deposited data**		

16S rRNA amplicon sequences	In this paper	BioProject: PRJEB68326

**Experimental models: Organisms/strains**		

C57BL/6J	The Jackson Laboratory	JAX stock #000664 RRID:IMSR_JAX:000664
Iga−/− (Ighatm1Grh)	SPF Vivarium LASC Schlieren (Harriman et al., 1999)	MGI: 1857185

**Recombinant DNA**		

NICE® pNZ8123 Lactococcus lactis secretion vector (SP Usp45/NaeI)	MoBiTec GmbH	Cat# VS-ELV00750-01

**Software and algorithms**		

FlowJo v10.7	https://www.flowjo.com/	RRID:SCR_008520
FACSDiva 9.1.2	BD Bioscience	RRID:SCR_001456
GraphPad Prism 10.1.0	https://www.graphpad.com	RRID:SCR_002798
NDP.view2 Viewing software U12388-01	Hamamatsu Photonics	N/A
FastQC 0.11.8		RRID:SCR_014583

### Resource availability

#### Lead contact

Further information and requests for resources and reagents should be directed to the lead contact Fabio Grassi (fabio.grassi@irb.usi.ch).

#### Materials availability

Materials generated in this study are available upon reasonable request and will be fulfilled under a material transfer agreement (MTA).

#### Data and code availability

16S rRNA sequencing files were submitted to the NCBI Sequence Read Archive (www.ncbi.nlm.nih.gov/sra) and are available at BioProject: https://www.ncbi.nlm.nih.gov/bioproject/PRJEB67750 PRJEB67750.

This paper does not report original code.

Any additional information required to reanalyze the data reported in this paper is available from the lead contact upon request.

### Experimental model and subject details

#### Mice

Specific pathogen-free (SPF) C57BL/6 and Iga^−/−^ (Igha^tm1Grh^) mice were bred at the Institute for Research in Biomedicine, Bellinzona, Switzerland. All animal experiments were performed in accordance with the Swiss Federal Veterinary Office guidelines and authorized by the Cantonal Veterinary (approval no. 31975 TI-19/2020/2023; 34029 TI-40/2021; 34591 TI-02/2022). Animals were supervised by a licensed veterinarian and proper steps were taken to ensure the welfare and minimize the suffering of all animals in the conducted studies. For all the experiments, mice were used immediately after birth or at 4–8 weeks of age. Mice were age- and sex-matched. Animals were housed in ventilated cages in a 12 h light/dark cycle, with free access to water and standard sterilized chow.

#### Intergenerational mouse model of undernutrition

At eight weeks of age, female C57B/6 or Iga^−/−^ mice were randomized into receiving either a malnourished diet moderately low in protein and fat (MAL: 7% protein and 5% fat), or an isocaloric conventional diet (CON: 20% protein and 15% fat) (Table S2). The chow was sterilized by irradiation before use and mice were given the diet *ad libitum* throughout experiments. After 14 days, CON and MAL female C57B/6 or Iga^−/−^ mice were mated with a male mouse. Pups born from both CON and MAL dams were daily monitored for their survival, body weight, tail length and behavior until 21 days after birth. Starting immediately after birth, MAL pups were orally gavaged with 10^8^ colony forming units (CFU) of *L. lactis*^pNZ^ or *L.lactis*^pNZ−Apyr^ two times a week until 21 days after birth. Pups were daily monitored for their body weight, tail length and general conditions until 21 days after birth.

#### Cholera toxin immunization

10 weeks-old MAL and CON mice, orally administered two times a week with *L. lactis*^pNZ^ or *L. lactis*^pNZ−Apyr^, were immunized by oral gavage with a 200 μL solution containing 10 μg of whole cholera toxin (CT) (C8052, Sigma Aldrich Chemie GmbH, Germany), and 10 μg of chicken ovalbumin (A5503, OVA, Sigma Aldrich Chemie GmbH, Germany) dissolved in 3% sodium bicarbonate, pH 8.0. Mice received three consecutive immunizations at 7-day intervals. Immunized mice were sacrificed 7 days post last immunization.

#### Breast milk collection

Breast milk (BM) was collected postmortem at 21dayays post-delivery. Prior to milk collection, dams were separated from their litters at least 2–4 h before milking to ensure higher quantity of milk collection. Breast milk was manually pressed from the dams’ teat by using thumb and forefinger to gently massage and squeeze the mammary tissue in an upward motion until a visible bead of milk begins to form at the base of the teat. The breast milk was then collected with a 10μL pipette tip. BM samples were stored at −20°C until use.

#### Collection of biosamples

For *ex vivo* experiments, mice were euthanized by CO_2_ inhalation, blood was collected directly from the heart using a syringe with a 26G needle, left for 1 h at room temperature and serum was separated by centrifugation (twice for 10 min at maximum speed) and stored at −80°C for further analysis. Afterward, the abdomen was carefully opened and small intestine, Peyer’s Patches (PPs) and colon were harvested and processed as described below. Small intestine and colon length were measured using a standard ruler (0.1 cm interval). Intestinal content was carefully removed from the small intestine, caecum, and colon using tweezers and aliquots stored at −80°C until further analysis.

#### FITC-dextran assay

Intestinal permeability was assessed using FITC-dextran assay. Mice were starved for 4h before treatment and then orally gavaged with 6 mg/mouse FITC-dextran (molecular weight 4000) (46944, Sigma Aldrich, Sweden) in PBS (D8537, Sigma Aldrich, UK). Blood was collected 4 h post-treatment, the serum samples were diluted 1:2 with PBS 25 mM HEPES (15630-056, Gibco, UK) and the fluorescence intensity was measured in the serum at 485/530 nm using a micro-plate reader (Biotek Synergy 2).

#### Bacterial strains and growth conditions

For the expression of Shigella flexneri apyrase in the *Lactococcus lactis* NZ900 strain, the apyrase encoding gene *phoN2* was PCR amplified from the *S. flexneri* genome and cloned into the pNZ8123 plasmid, generating the pNZ-Apyr plasmid (Figure S4A). Apyrase expression in the pNZ-Apyr plasmid is controlled by the PnisA promoter, which is inducible by the nisin anti-microbial peptide. The *phoN2* gene was in-frame cloned with the signal sequence of the L. lactis major secreted protein Usp45 to allow apyrase secretion. *L. lactis*^pNZ^ and *L. lactis*^pNZ−Apyr^ strains were grown in M17 medium (CM0817B, Merk, Germany) supplemented with glucose (0.5% w/v, G7021-1KG, Sigma Aldrich, USA) and nisin (4 ng/mL, N5764-5G, Sigma Aldrich, USA).

For calculation of CFU in the mesenteric lymph nodes (MLN), CON and MAL mice were sacrificed at the end of the experiment, MLN were harvested aseptically into RPMI (42401-018, Gibco, UK) and mechanically homogenized. Dilutions of homogenates were plated onto Schaedler agar (91019, Sigma Aldrich, Millipore, India). Plates were grown under aerobic or anaerobic culture conditions.

### Method details

#### Cells purification and flow cytometry

Immunophenotyping of gut adaptive immune system was analyzed in 21 days old mice born from WN or MN mothers and in WN and MN dams after delivery. At sacrifice, PPs were collected and homogenized in RPMI to obtain single cells suspension. The whole small intestine and colon were delicately separated by faces and opened longitudinally to be further processed. Small intestine and colon were washed twice with ice-cold PBS and digested at 37°C for 30 min with RPMI (42401-018, Gibco, UK) added with 5 mM EDTA (A3145, AppliChem, Germany) for two times. The filtrated fragments were then digested in RPMI 5%, heat-inactivated fetal bovine serum (FBS) (A5256701, Gibco, Mexico), 1 mg/mL collagenase from Clostridium histolyticum (C2139, Sigma Aldrich, Israel) and 40 μg/mL DNase-I grade II (4851, Roche Diagnostics, Germany) for 40 min. The filtered suspension, containing the lamina propria cells, was centrifuged for 5 min at 1500 rpm and resuspended in 40% Percoll (17089101, Cytiva, Sweden). 80% Percoll was delicately added to samples using a Pasteur pipette, which were then centrifuged for 20 min at 2000 rpm, at room temperature without acceleration and brake. The leukocytes ring formed at the interface between 40% and 80% Percoll was collected, resuspended in FBS, homogenized, centrifuged for 5 min at 1500 rpm and finally resuspended in RPMI complete medium. Single-cell suspensions from Peyer’s patches, small intestine and colon lamina propria were stained with anti-mouse labeled antibodies diluted in PBS with 2% heat-inactivated FBS for 20 min on ice.

##### SI and colon regulatory T cells (Tregs)

For the analysis of Treg cells, single-cell suspensions of small intestine and colon lamina propria were first stained extracellularly using APCCy7 conjugated anti-CD3ε (1:200) (Clone: 145-2C11, Cat: 100330, BioLegend), PECy7 conjugated anti-CD4 (1:200) (Clone: GK 1.5, Cat: 100422, BioLegend), PE conjugated anti-CD25 (1:200) (Clone: PC61, Cat: 102007, BioLegend), PerCP/Cyanine5.5 conjugated anti-ICOS (1:200) (Clone: C398.4A, Cat: 313518, BioLegend), PB conjugated anti-CD8α (1:200) (Clone: 53-6.7, Cat: 100725, BioLegend), FITC conjugated anti-CD45 (1:400) (Clone: 30-F11, Cat: 103108, BioLegend). Cells were then intracellularly stained with APC conjugated anti-FOXP3 (1:100) (Clone: FJK-16s, Cat: 17-5773-82, Invitrogen).

##### SI lamina propria plasma cells (SILP PCs)

For the analysis of plasmacells, single-cell suspensions of small intestine lamina propria were stained extracellularly using PB conjugated anti-B220 (1:200) (Clone: RA3-6B2, Cat: 103227, BioLegend), PE conjugated anti-CD45 (1:400) (Clone: 30-F11, Cat: 103106, BioLegend), APCCy7 conjugated anti-CD19 (1:200) (Clone: 6D5, Cat: 115530, BioLegend), FITC conjugated anti-IgA (1:200) (PRID: AB_2794370 Cat: 1040-02, Southern Biotech), PECy7 conjugated anti-IgG (1:200) (clone: Poly4053; RRID: AB_10662421 Cat: 45315, BioLegend) and APC conjugated anti-TCRb (1:200) (Clone: H57-597, Cat: 109212, BioLegend).

##### PPs germinal center B cells (GC B cells)

For the analysis of germinal center B cells, single-cell suspensions of Peyer’s patches were stained extracellularly with APCCy7 conjugated anti-CD19 (1:200), PB conjugated anti-B220 (1:200), FITC conjugated anti-PNA (1:500) (Cat: FL-1071, Vector Laboratories, Inc.) and PE conjugated ant-FAS (CD95) (1:200) (Cat: 554258, BD Biosciences).

##### PPs T follicular helper cells (Tfh), T follicular helper cells (Tfh) and T regulatory cells (Treg)

For the analysis of Tfh, Tfr and Treg cells, single-cell suspensions of Peyer’s patches were first stained extracellularly with APC conjugated anti-TCRb (1:200), APCCy7 conjugated anti-CD4 (1:200) (Clone: RM4-5, Cat: 100526, BioLegend), PECy7 conjugated anti-CD25 (1:200) (Clone: PC61, Cat. 102016, BioLegend), PE conjugated anti-ICOS (1:200) (Clone: 15P9, Cat: 107706, BioLegend), Biotinylated anti-CXCR5 (1:50) (Cat: 551960, BD Biosciences) and PerCP/Cyanine5.5 conjugated anti-PD1 (1:200) (Clone: RMP1-30, Cat: 109120, BioLegend). PB coniugated streptavidin was used (1:100) (eF450, Cat: 48-4317-82, eBioscence). Cells were also stained intracellularly with FITC conjugated anti-FOXP3 (1:100) (Clone: FJK-16s, Cat: 11-5773-82, eBioscience, Inc).

Samples were acquired on an LSR Fortessa (BD Biosciences, Franklin Lakes NJ, USA) flow cytometer. Data were analyzed using the FlowJo software (TreeStar, Ashland, OR, USA) or FACS Diva software (BD Biosciences, Franklin Lakes NJ, USA).

##### Colon infiltrating monocytes

For the detection of CD45^+^CD11b^+^Ly6G^−^Ly6C^+^ monocytes, colon single-cell suspensions was stained with FITC conjugated anti-CD45 (1:400) (Clone: 30-F11, Cat: 103108, Biolegend), PECy7 conjugated anti-CD11b (Clone: M1/70, Cat: 101216, BioLegend), PB conjugated anti-Ly6G (Clone: 1A8, Cat: 127612, BioLegend) and Biotinylated anti-Ly6C (Clone: HK1.4, Cat: 128004, BioLegend), PerCP conjugated streptavidin (1:200) (Cat: 405213, BioLegend)

##### Colon infiltrating neutrophils

For the detection of CD45^+^CD11b^+^Ly6G^+^Ly6C^+^ neutrophils, colon single-cell suspensions was stained with FITC conjugated anti-CD45 (1:400) (Clone: 30-F11, Cat: 103108, Biolegend), APC conjugated anti-CD11c (Clone: N418, Cat: 17-0114-82, eBioscience), PECy7 conjugated anti-CD11b (Clone: M1/70, Cat: 101216, BioLegend), and PB conjugated anti-Ly6G (Clone: 1A8, Cat: 127612, BioLegend), and gated as illustrated in Figure S1A.

##### IgA coated bacteria

For analysis of IgA coated bacteria in flow cytometry, fresh fecal pellets were collected into sterile 2mL tubes and homogenized in PBS (0.1 g/mL). The homogenized samples were centrifuged at 400 g for 5 min to remove large debris. Supernatants were centrifuged at 8ʼ000g for 10 min to remove unbound IgAs. Bacterial pellets were resuspended in PBS 5% goat serum (G9023, Sigma Aldrich Chemie GmbH, Germany), incubated 15 min on ice, centrifuged and resuspended in PBS 1% bovine serum albumin (BSA) (A7906, Sigma Aldrich, USA) for staining with APC conjugated rabbit anti-mouse IgA antibodies (Clone: mA-6E1, Cat: 17-4204-82, Invitrogen). After 30 min incubation, bacteria were washed twice and resuspended in 2% paraformaldehyde (CAS 30525-89-4 | 818715, Merck, US) in PBS for acquisition at LSRFortessa. For analysis, forward and side scatter parameters were used in logarithmic mode. SYTO BC Green Fluorescent Nucleic Acid (S34855, Invitrogen, USA) was added to identify bacteria-sized particles containing nucleic acids. Samples were acquired on an LSR Fortessa (BD Biosciences, Franklin Lakes NJ, USA) flow cytometer. Data were analyzed using the FlowJo software (TreeStar, Ashland, OR, USA) or FACS Diva software (BD Biosciences, Franklin Lakes NJ, USA).

#### Histological evaluation of small intestine and colon

Small intestine and colon from CON and MAL mice were examined at necropsy, fixed in 10% neutral buffered formalin for at least 48 h prior to embedding in paraffin, and stained with hematoxylin and eosin (H&E). Pathological scores in the small intestine were determined in a blinded manner using a previously described scoring scheme.^67^ Briefly, each tissue section was assessed for (1) inflammatory infiltrate (0, no change; 1, mild; 2, moderate; 3, marked; 4 marked, transmural) and (2) intestinal architecture (0, no change; 1, mild villous blunting; 2, moderate villous blunting and mild epithelial hyperplasia; 3, moderate villous blunting and broadening as well as moderate epithelial hyperplasia and goblet cell loss; 4, villous atrophy and ulcerations as well as marked epithelial hyperplasia and goblet cell loss). A maximum score under this scale is 8. Furthermore, PPs, villus and crypt length were measured using the NDP.view2 image viewing software U12388-01 from Hamamatsu (Hamamatsu Photonics France S.A.R.L., Massy Cedex, France). In every intestine (duodenal, jejunal and ileal section), 10 villi and 10 adjacent crypts were evaluated in the scanned slide. Pathological scores in the colon were determined in a blinded manner using a previously described scoring scheme.^68^ Briefly, each tissue section was assessed for (1) submucosal edema (0, no change; 1, mild; 2, moderate; 3, severe); (2) crypt hyperplasia (0, no change; 1, 1–50%; 2, 51–100%; 3, >100%); (3) goblet cell depletion (0, no change; 1, mild depletion; 2, severe depletion; 3, absence of goblet cells); (4) epithelial integrity (0, no pathological changes detectable; 1, epithelial desquamation (few cells sloughed, surface rippled); 2, erosion of epithelial surface (epithelial surface rippled, damaged); 3, epithelial surface severely disrupted/damaged, large amounts of cell sloughing; 4, ulceration [with an additional score of 1 added for each 25% fraction of tissue in the cross-section affected up to a maximum score of 8 (4 + 4) for a tissue section that had entirely lost its crypt structure due to epithelial cell loss and immune cell infiltration); (5) mucosal mononuclear cell infiltration (per 400× magnification field) (0, no change; 1, <20; 2, 20–50; 3, >50 cells/field); (6) submucosal PMN and mononuclear cell infiltration (per 400× magnification field) (1, <5; 2, 21–60; 3, 61–100; 4, >100 cells/field). A maximum score under this scale is 24.

For PPs immunohistochemistry (IH), consecutive sections (3–5 μm) of the small intestine were used. IH was performed using the horseradish peroxidase (HRP) method to detect T cells (CD3^+^) and B cells (CD45R/B220^+^) in the ileal PPs. Antibodies and detection systems are listed in STAR Methods. Briefly, after deparaffination, sections underwent antigen retrieval in citrate buffer pH 6 at 96°C for CD45R or CC1 (Discovery, Roche Diagnostics, Switzerland) for CD3ε, and then incubated for 1 h at 37°C with the primary antibodies. This was followed by the blocking of endogenous peroxidase and incubation with the appropriate secondary antibodies/detection systems (Dako Agilent, Denmark for CD45R or Discovery, Roche Diagnostics, Switzerland for CD3).

#### Quantification of milk protein concentration

Breast milk total protein concentration was measured by Bradford protein assay. The samples were pre-diluted 1:100 in PBS. 1 μL of each sample was then diluted in 1mL of Comassie PlusTM Protein Assay Reagent (23236, Thermo Scientific) and incubated for 1 min in the dark. Absorbance was detected at 595 nm. To the exact protein concentration, we ran a standard curve using serial dilutions of known amount of Bovine Serum Albumin (BSA) (A7906, Sigma Aldrich, USA).

#### ELISPOT assay

Frequency of small intestine lamina propria secreting IgA plasmacells were detected using an ELISPOT assay. Briefly, 96-well plates (MSIPS4510, Millipore, Ireland) were coated with 5 mg/mL purified goat anti-mouse IgA (RRID:AB_2314669, Cat: 0106-01, Southern Biotechnologies) or Cholera Toxin (CT) antigen (C8052, Sigma Aldrich Chemie GmbH, Germany). After washing and blocking with1% BSA in PBS for 30 min, serial dilutions of total small intestine lamina propria cells were added and incubated at 37°C for 16 h. The plates were washed and incubated with anti-IgA biotin-conjugated secondary antibodies (Cat: 1040-08, Southern Biotech), followed by streptavidin horseradish peroxidase conjugate (HRP) (S911, Invitrogen, USA). The assay was developed with AEC (Sigma-Aldrich). Spot counting was performed using C.T.L. S6 Ultra-V Analyzer Immunospot.

#### ELISA assay

Small intestine, caecum and fecal samples were collected and processed. Briefly, fresh fecal pellets were collected into sterile 1.5 mL Eppendorf tubes and homogenized in PBS (0.1 g/mL). The samples were then centrifuged for 10 min at maximum speed. The centrifugation process was repeated for two consecutive times and supernatant was stored at −20°C until use. Breast milk samples were collected and processed as described above. Fecal IgA, breast milk IgA, IgM, IgG, IgG subclasses (IgG1, IgG2c, IgG2b, IgG3) and anti-cholera toxin (CT) specific IgA levels were detected by ELISA. Briefly, ELISA plates (3690, half-area 96 well plate, Corning, USA) were coated (16 h at 4°C) with 25 μL of unlabeled goat anti-murine IgA (Cat: 1040-01), IgM (Cat: 101-01), IgG (Cat: 1031-01), IgG1 (Cat: 1070-01), IgG2c (Cat: 1079-01), IgG2b (Cat: 1090-01), IgG3 (Cat: 1100-01) (all from Southern Biotech) or CT antigen (Cat: C8052 Sigma-Aldrich, UK) at 5 μg/mL in PBS, washed 4 times with PBS 0.025% Tween 20 (Cat: 93773, Sigma Aldrich, UK) and saturated with 50 μL of PBS 1% BSA for 1 h at room temperature. Twenty-five μL of serial dilutions of the different samples were incubated 2 h at room temperature. After 4 washes in PBS 0.025% Tween 20, 25 μL of alkaline phosphatase (AP) conjugated goat anti-mouse IgA (Cat: 1040-04), IgM (Cat: 1021-04), IgG (Cat: 1030-04), IgG1 (Cat: 1070-04), IgG2c (Cat: 1079-04), IgG2b (Cat. 1090-04) or IgG3 (Cat: 1100-04) (all from Southern Biotech) (1:500 in PBS 1% BSA) were added and plates were incubated for 2h at room temperature. The assay was developed with 4-Nitrophenyl Phosphate disodium salt hexahydrate (Cat: N2765-100TAB, Sigma Aldrich) in carbonate buffer, composed by 1.59 g Na_2_CO_3_/L deionized water (Cat: 1.06395.0500, Merk, Germany) and 2.93 g NaHCO3/L deionized water (Cat: A0384,0500, AppliChem, Germany), and absorbance was detected at 405nm. The CT-IgA ratio was calculated as a ratio of cholera-specific IgA (defined by OD450nm) to total IgA (expressed in μg/mL).

#### Lipocalin-2 quantification

The inflammation status of mice was evaluated by measuring the levels of Lipocalin-2 (LCN-2) in fecal supernatants via ELISA assay (DY1857, R&D systems, DuoSet ELISA Mouse Lipocalin-2/NGAL). Briefly, fresh fecal pellets were collected into sterile 1.5 mL Eppendorf tubes and homogenized in PBS (0.1 g/mL). The samples were then centrifuged twice for 10 min at maximum speed and the supernatant was stored at −20°C until use. Adequate serial dilutions of fecal samples were used to perform the LCN-2 ELISA assay following the manufacturer’s suggestion (https://www.rndsystems.com/products/mouse-lipocalin-2-ngal-duoset-elisa_dy1857#assay-procedure). Absorbance was read at 405 nm and the LCN-2 concentration obtained was normalized for the dilution factor and for stools weight.

#### Quantification of ATP

For quantification of ileal ATP after oral treatment with *L. lactis*^pNZ^ or *L.lactis*^pNZ−Apyr^, intestinal content was collected by lavage with 10 mL of intestinal wash buffer, composed by PBS, 0.5 M EDTA, Soybean trypsin inhibitor (Cat: T6522, Sigma Aldrich, USA) and phenylmethanesulfonyl fluoride (PMSF) (Cat: P7625, Sigma Aldrich, USA), spun and filtered (0.22 μm) (SLGP033RS, Merk, Ireland) to remove any bacteria-sized contaminants and immediately frozen in dry ice. ATP concentration in the intestinal washes was multiplied for the dilution factor to obtain the actual endoluminal ATP concentration, as previously described.^51^ The extracellular ATP concentration was evaluated by bioluminescence assay (ATP determination Kit, A22066, Invitrogen) with recombinant firefly luciferase and its substrate D-luciferin according to the manufacturerʼs protocol (https://assets.thermofisher.com/TFS-Assets%2FLSG%2Fmanuals%2Fmp22066.pdf).

#### 16S rRNA gene bacterial profiling

Extraction, lysis, and DNA isolation were carried out utilizing the Fast DNA Stool Mini Kit (Qiagen) in strict accordance with the manufacturer’s recommendations. Bead beating was performed using a FastPrep24 instrument (MPBiomedicals), with four cycles of 45 s each at a speed of 4. The bead beating process was conducted in 2 mL screw-cap tubes containing 0.6 g of 0.1 mm glass beads. A total of 200 mL of raw extract was prepared for subsequent DNA isolation. To assess the concentration of the isolated DNA, we employed PicoGreen measurement, employing the Quant-iTT PicoGreenT dsDNA Assay Kit from Thermo Fisher. Furthermore, to verify the integrity of a representative sample, agarose gel electrophoresis was conducted. For the amplification of the bacterial 16S rRNA gene, a specific primer set targeting the V3–V4 hypervariable regions was employed. The forward primer (Fw) utilized in this study was 5′-CCT ACG GGN GGC WGC AG-3' (SEQ ID NO: 4), and the reverse primer (Rev) was 5′-GAC TAC HVG GGT ATC TAA TCC-3' (SEQ ID NO: 5).

### Quantification and statistical analysis

#### Bioinformatics of 16S rRNA gene sequencing data

Sequencing of the PCR libraries was performed on the Illumina MiSeq platform using a v2 500 cycles kit. The generated paired-end reads, which successfully passed Illumina’s chastity filter, underwent de-multiplexing and the removal of Illumina adapter remnants. These processes were executed using Illumina’s real-time analysis software integrated into the MiSeq reporter software v2.6. No further post-processing or selection was applied at this stage. To assess the quality of the reads, FastQC version 0.11.8 software was employed. The initial dataset consisted of a total of 2,719,572 sequences (with a median read count of 23,776 and a mean of 34,866). Subsequent trimming of the first 7 and last 25 bases, as well as reads filtration, led to the generation of sequences with excellent quality (Phred >30). These high-quality sequences, totaling 1,960,391 (with a median read count of 17,013 and a mean of 25,133), were subjected to a denoising algorithm.^69^ This algorithm effectively merged the overlapping regions R1 and R2, while eliminating chimeric reads. Taxonomic assignment was carried out using the BLAST feature-classifier, which performed local alignment via BLAST+ between query and reference reads. The consensus taxonomy for each query sequence was assigned based on the latest Greengenes database version (gg_12_10). For phylogenetic analysis, a rooted tree was constructed utilizing the IQ-TREE stochastic algorithm, facilitating maximum likelihood analysis of extensive phylogenetic data.^70^

#### Statistics

Statistical analysis for 16S rRNA gene sequencing data was performed as described above. Experimental group comparisons were performed by applying the nonparametric Mann-Whitney-U (MWU) test. To test for differences between the developments of the body weight over time in the offspring, linear regression models were built and compared by employing an ANOVA. *p*-values of <0.05 were judged to be significant (∗*p* < 0.05, ∗∗*p* < 0.01, ∗∗∗*p* < 0.005, ∗∗∗∗*p* < 0.001). Statistical analyses were conducted using GraphPad Prism version 9.5.1. To discern potential differences in microbiota composition between the two groups of subjects (CON, MAL) and (MAL, MAL pups treated with *L. Lactis*^pNZL^ or *L. Lactis*^*pNZ-Apyr*^), differential analysis was carried out. The LEfSe algorithm (https://doi.org/10.1186/gb-2011-12-6-r60) was employed for this purpose, allowing for the identification of specific bacterial taxa that could serve as discriminatory markers between the groups. Alpha diversity was calculated using the main indexes to allow an exploration of data in term of richness and evenness. Alpha-diversity estimates were computed using the phyloseq R package.^71^ Statistically significant changes in the alpha diversity were determined through the Mann-Whitney signed-rank test. The microbial community comparison was calculated using PERMANOVA on Weighted distance metrics performed by UniFrac algorithm.^72^ Statistically significant differences in the relative abundance of ASVs between groups were performed by Wald test using FDR *p* value correction following DESeq2 read counts normalization.

## Supp;Supplemental information

Document S1. Figures S1–S6

Table S1. Taxonomic differences between CON and MAL pups, related to Figure 3

Table S2. Composition of CON and MAL diets, related to Figure 1

Document S2. Article plus supplemental information
