# Supplementary material for: Protection from environmental enteric dysfunction and growth improvement in malnourished newborns by amplification of secretory IgA
Source: Cell Rep Med. 2024 Jul 2;5(7):101639. doi: 10.1016/j.xcrm.2024.101639 (PMC11293325; doi:10.1016/j.xcrm.2024.101639)
Supplement: Document S1. Figures S1–S6 [file mmc1.pdf]

Cell Reports Medicine, Volume *5*

Supplemental information

Protection from environmental enteric dysfunction and growth improvement in malnourished newborns by amplification of secretory IgA

Lisa Perruzza, Tanja Rezzonico Jost, Matteo Raneri, Giorgio Gargari, Martina Palatella, Benedetta De Ponte Conti, Frauke Seehusen, Julia Heckmann, Dorothee Viemann, Simone Guglielmetti, and Fabio Grassi

Supplementary Figures

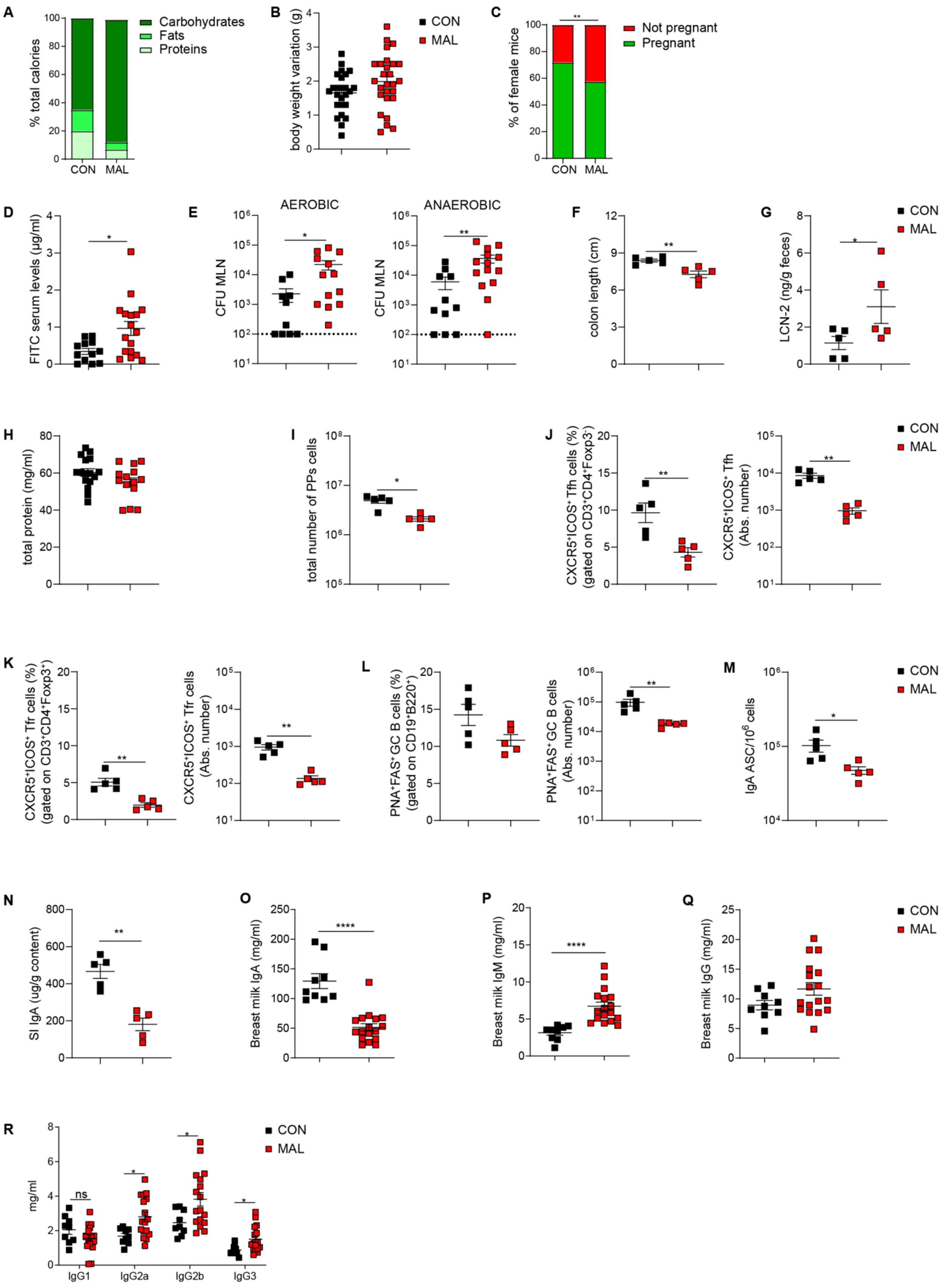

Supplementary Figure S1. Effect of Malnutrition on the parental mice, related to Figures 1 and 2.

Assessment of malnutrition's effects on body weight variation, fertility, intestinal barrier function and mucosal adaptive immunity in 8 weeks old C57BL/6 female mice.
(A) Schematic of the components of each diet expressed as a percentage of total calories.
(B) Body weight variation in CON and MAL mice. Starting from two weeks prior to mating, 8 weeks old female mice were fed with CON or MAL diet. Body weight variation after 15 days of treatment is shown.
(C) A schematic showing the frequency of pregnancy expressed as percentage of pregnant or not pregnant CON (N = 146) or MAL (N = 250) female C57BL/6 mice. Chi-square test was used. $^{**}p < 0.01$.
(D) Concentration of FITC in the serum was assessed 4 h post dextran-FITC oral administration, after mice were fed each diet for 5 weeks (n = 12-17 mice per group, pooled data from independent experiments, Mann-Whitney U-test).
(E) CFU quantification of aerobic and anaerobic bacteria recovered from the MLN (n = 10-12 mice per group, pooled data from independent experiments, Mann-Whitney U-test).
(F-G) Measurement of colon length (cm) (F) and fecal LCN-2 concentration (ng/g feces) (G) in CON and MAL mice (n = 5 mice per group, one representative experiment out of three is shown, Mann-Whitney U-test).
(H) Total milk protein concentrations (mg/mL) quantified by Bradford assay (n = 13-14 mice per group, pooled data from independent experiments, Mann-Whitney U-test).
(I) PPs cellularity expressed as total number of PPs cells in CON and MAL mice.
(J-L) Statistical analysis and relative absolute number of Tfh (J), Tfr (K), and GC B (L) cells in PPs from CON and MAL mice.
(M) IgA secreting plasma cells measured by ELISPOT assay in the lamina propria of the small intestine from CON and MAL mice.
(N) Quantification of total IgA by ELISA in the small intestine of CON and MAL mice. (n = 5 mice per group, one representative experiment out of three is shown, Mann-Whitney U-test).
(O-R) ELISA quantification of total IgA (O), IgM (P), IgG (Q), and IgG1, IgG2c, IgG2b and IgG3 (R) concentration (mg/ml) in CON and MAL dams 21 days after delivery (n = 10-15 mice per group, pooled data from independent experiments, Mann-Whitney U-test).
$^{*}p < 0.05$; $^{**}p < 0.01$; $^{****}p < 0.0001$; ns: not significant.

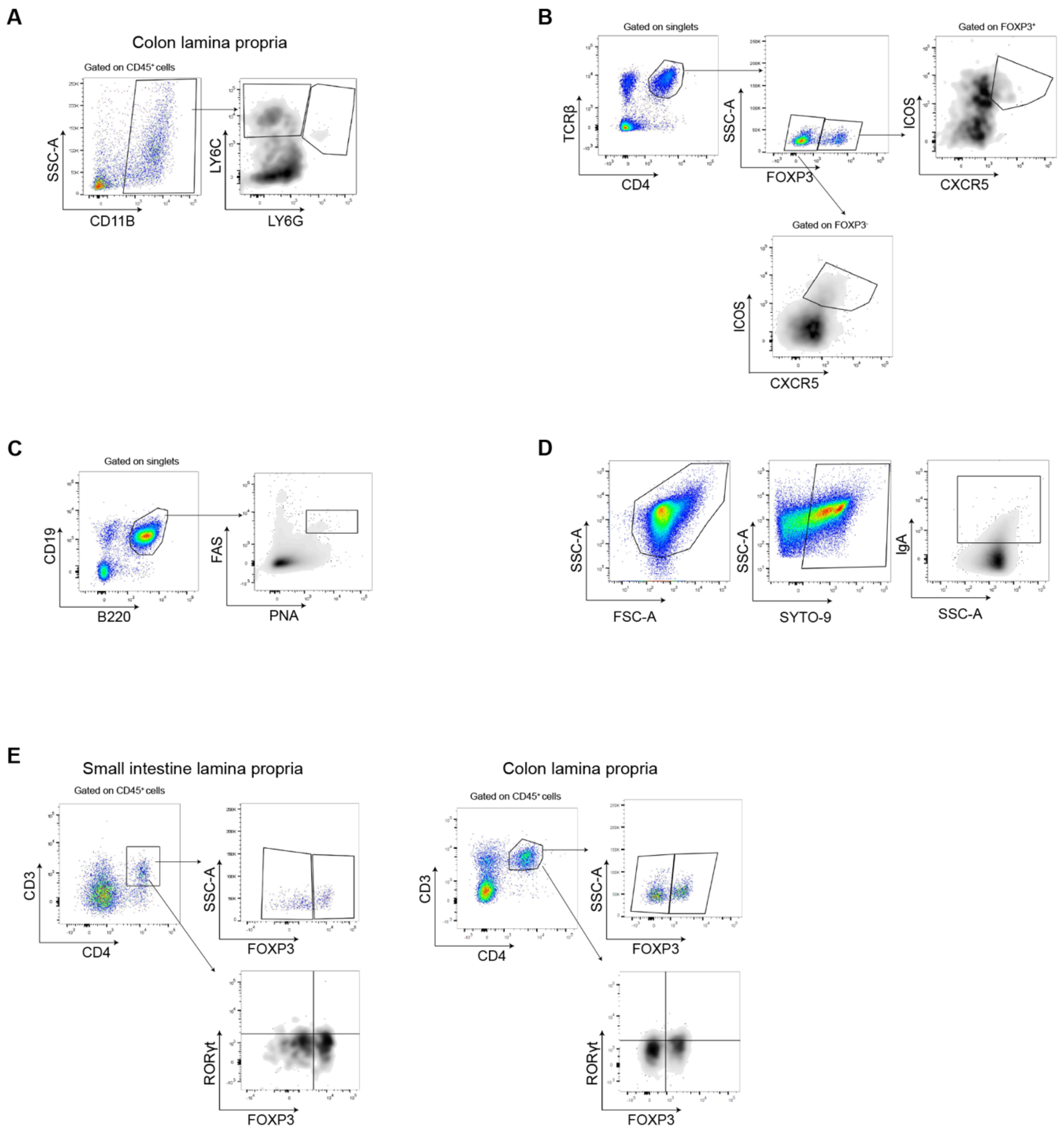

Supplementary Figure S2. Gating strategy for the analysis of different cell subsets and bacteria in flow cytometry, related to Figures 1 and 2.

Immune system cells and bacteria were isolated and stained as described in the Methods section. Gating strategy for the analysis of:

(A) colon lamina propria monocytes (CD11b$^+$Ly6C$^+$Ly6G$^-$) and neutrophils (CD11b$^+$Ly6C$^+$Ly6G$^+$).

(B**)** Tfh (Foxp3$^-$ICOS$^+$CXCR5$^+$) and Tfr (Foxp3$^+$ICOS$^+$CXCR5$^+$) cells isolated from PPs.

(C**)** GC B (CD19$^+$B220$^+$Fas$^+$PNA$^+$) cells isolated from PPs.

(D**)** IgA coated bacteria from the stools

(E**)** total Foxp3$^+$ or Foxp3$^+$RORγ$^+$ Treg cells from small intestine and colon lamina propria.

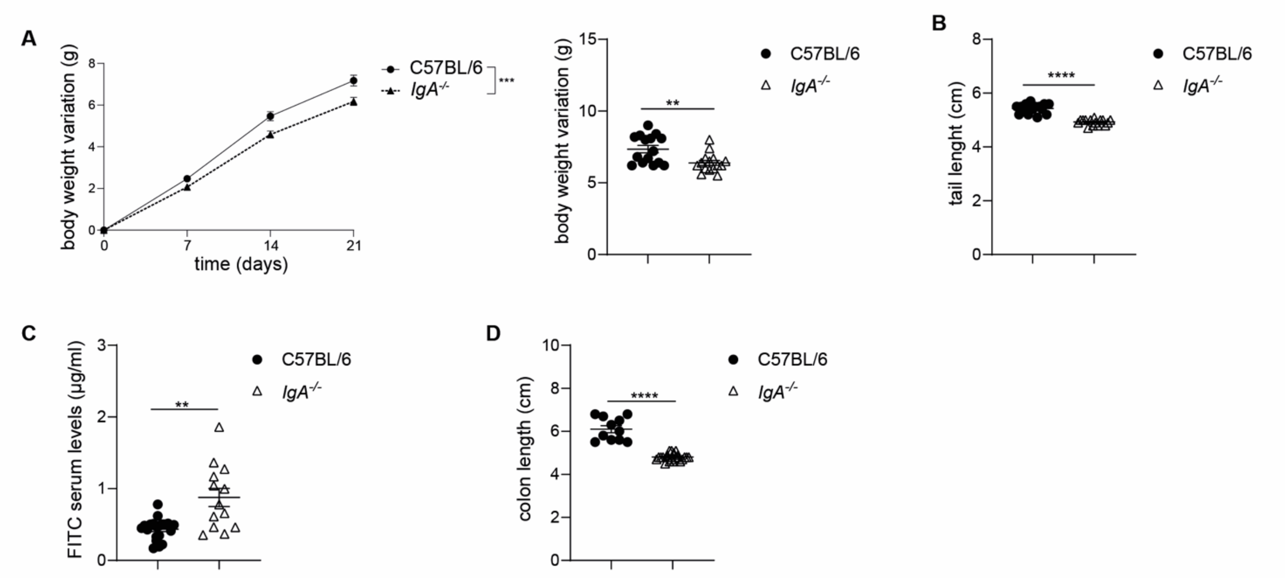

Supplementary Figure S3. Role of IgA in neonatal growth and intestinal homeostasis, related to Figures 1 and 2.

(A) Body weight variation over 21 days after birth (left) and body weight at 21 days after birth (right) in C57BL/6 and *IgA*$^{-/-}$ CON mice (n = 10-15 mice per group, pooled data from independent experiments, two-way ANOVA, left and Mann-Whitney U-test, right).

(B) Measurement of tail length (cm) 21 days after birth in C57BL/6 and *IgA*$^{-/-}$ CON mice (n = 10-15 mice per group, pooled data from independent experiments, Mann-Whitney U-test).

(C) Concentration of FITC in the serum was assessed 4 h post oral administration of FITC-dextran, in C57BL/6 and *IgA*$^{-/-}$ CON mice, 21 days after birth (n = 10-15 mice per group, pooled data from independent experiments, Mann-Whitney U-test).

(D) Colon lengths (cm) in C57BL/6 and *IgA*$^{-/-}$ CON mice, 21 days after birth (n = 10-15 mice per group, pooled data from independent experiments, Mann-Whitney U-test). Data points represent individual mice, Mean ± SEM are shown.

**$p < 0.01$; ***$p < 0.001$; ****$p < 0.0001$.

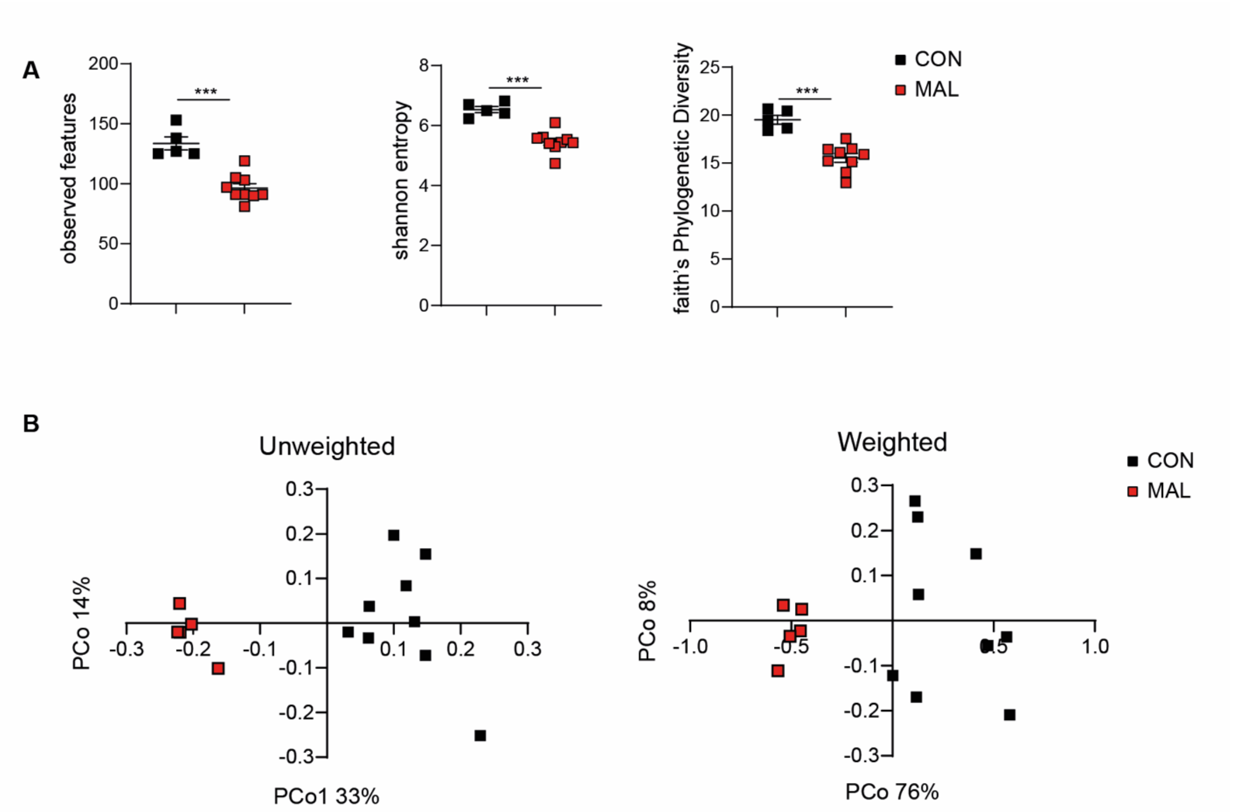

Supplementary Figure S4. Microbiota characterization in CON and MAL mothers, related to Figure 3.

(A) Bacterial α-diversity calculated by Observed features, Shannon index and Faith's phylogenetic diversity in CON and MAL mothers. (Two-tailed Mann-Whitney U-test). Data points represent individual mice (n = 5-9). Mean ± SEM are shown. ***$p < 0.001$.

(B) Bacterial β-diversity. The PCoA plots of microbial β-diversity were generated using Unweighted and Weighted UniFrac algorithms. PERMANOVA was used. $p < 0.001$ (n = 5-9 mice per group). Data points represent individual mice.

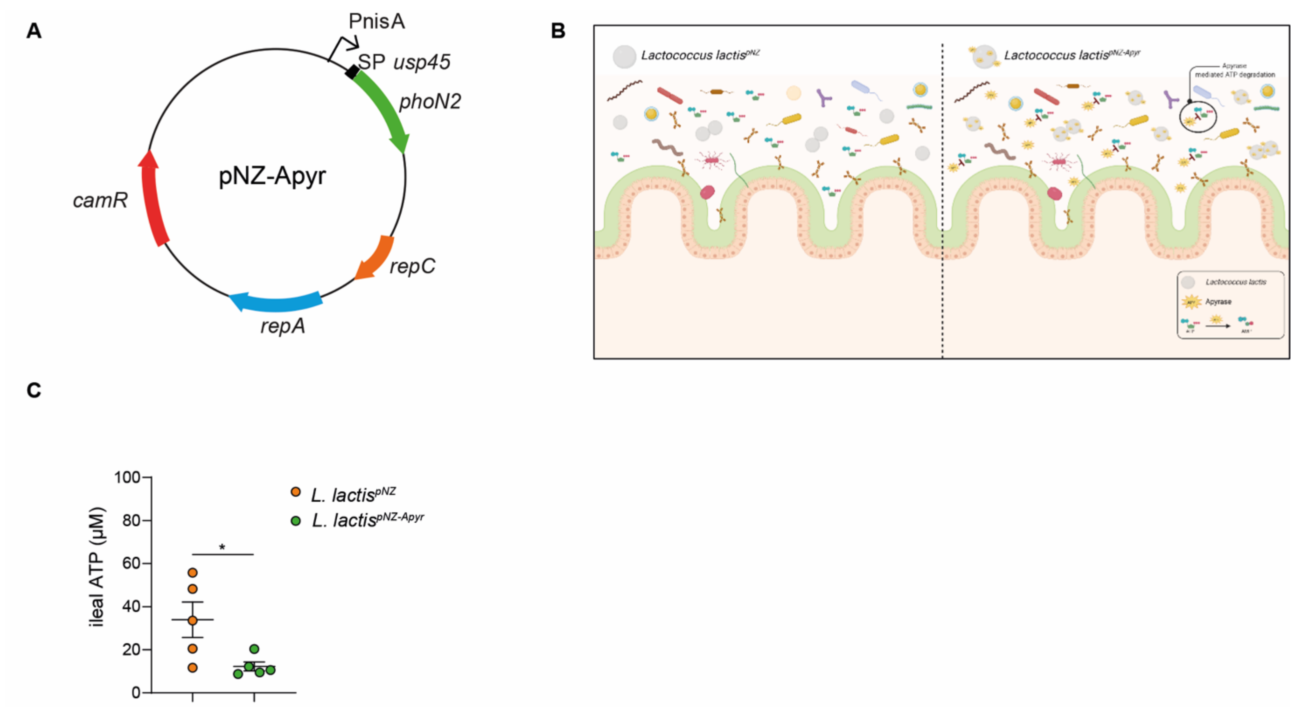

Supplementary Figure S5. Design and production of apyrase expressing *Lactococcus lactis*, related to Figure 4.
(A) Map of the pNZ-Apyr plasmid carrying the *phoN2* gene encoding apyrase used to transform *Lactococcus lactis*. P_{nisA}, *nisin A* inducible promoter; SP *usp45*, signal sequence of *usp45* gene; *phoN2*, *S. flexneri* apyrase gene; *repC*, replication gene C; *repA*, replication gene A; *camR* (*cat*), chloramphenicol resistance gene.
(B) Schematic representation of small intestine colonization by *L. Lactis* ^{pNZ} (left) and *L. Lactis*$^{pNZ\text{-}Apyr}$ (right) and related ATP degradation.
(C) Quantification of ileal ATP in mice colonized with *L. Lactis* ^{pNZ} or *L. Lactis*$^{pNZ\text{-}Apyr}$ ($n = 5$ mice per group, one representative experiment out of three is shown), Mann-Whitney U-test. *$p < 0.05$.

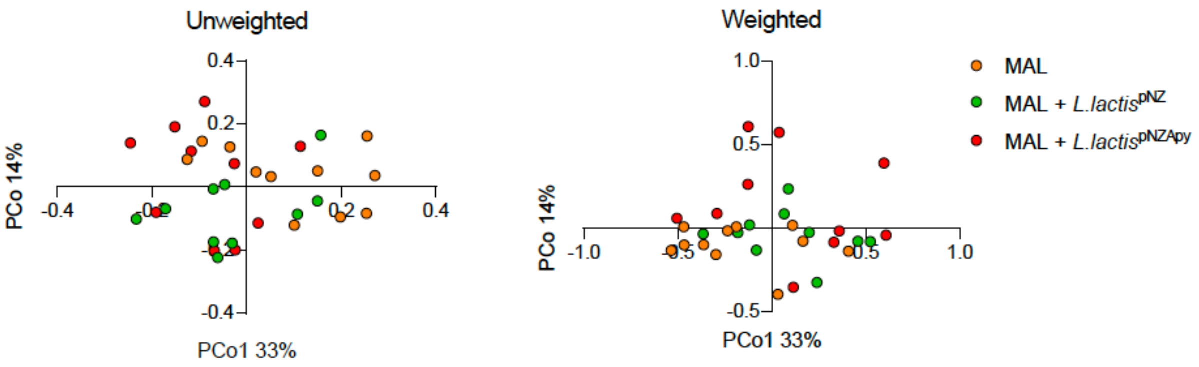

Supplementary Figure S6. Effect of apyrase on the pups' microbiota diversity, related to Figure 6.

Bacterial β-diversity in MAL pups and MAL pups treated with *L. Lactis*^{pNZ} or *L. Lactis*$^{pNZ\text{-}Apyr}$. The PCoA plots of microbial β-diversity were generated using Unweighted and Weighted UniFrac algorithms. PERMANOVA was used. $p < 0.001$ (n = 10-11 mice per group). Data points represent individual mice.
